# Lactobacilli-host interactions inhibit *Staphylococcus aureus* and *Escherichia coli*-induced cell death and invasion in a cellular model of infection

**DOI:** 10.3389/fmicb.2024.1501119

**Published:** 2024-12-18

**Authors:** Despoina Eugenia Kiousi, Maria Panopoulou, Aglaia Pappa, Alex Galanis

**Affiliations:** ^1^Department of Molecular Biology and Genetics, Democritus University of Thrace, Alexandroupolis, Greece; ^2^Department of Medicine, Faculty of Health Sciences, Democritus University of Thrace, Alexandroupolis, Greece

**Keywords:** *Lactiplantibacillus plantarum*, probiotics, pathogen-induced cytotoxicity, pathogen invasion, competitive exclusion, dual RNA-seq, transcriptomics, proteomics

## Abstract

Probiotic lactobacilli exert antimicrobial and antibiofilm activity through a plethora of mechanisms, including the production of inhibitory compounds and competition for nutrients and adhesion sites on the host mucosa. Their ability to interfere with pathogen-host interactions may mitigate the harmful effects of infection. *Lactiplantibacillus pentosus* L33 (L33) and *Lactiplantibacillus plantarum* L125 (L125) are two potential probiotic lactic acid bacteria (LAB) strains, previously shown to reduce pathogen viability and biofilm formation *in vitro*. The present study aims to investigate their ability to limit cell death induced by *Staphylococcus aureus* and *Escherichia coli* in the human colon adenocarcinoma cell line HT-29. To this end, we examined the protective effects of the two strains using *in silico*, *in vitro* and omic approaches, with *Lacticaseibacillus rhamnosus* GG (LGG) serving as a reference strain, due to its well-documented antimicrobial properties. Based on the findings of our study, direct contact of HT-29 cells with L125 for 4 h prior to the addition of *S. aureus* or *E. coli* prevented pathogen-induced cell death at rates comparable to LGG. In contrast, L33 failed to exert a protective effect. Moreover, L125 significantly reduced adherence of *S. aureus* to HT-29 cells, and the internalization capacity of both pathogens (>1.5 Log CFU/mL). Dual RNA-seq and protein microarrays were used to determine expression changes in L125 and host cells during co-incubation. L125 expressed high levels of adhesins and moonlighting proteins, homologous to those encoded by the pathogens. Pathways involved in pathogen adhesion and internalization, endocytosis, cell–cell and cell-extracellular matrix (ECM) adhesion, were downregulated in HT-29 cells. Finally, L125 reduced the secretion of various pro-inflammatory mediators. Our findings highlight the strain-specific protective effects of LAB against pathogen-induced cell death achieved through competitive exclusion and priming of host cell responses. Future studies will focus on elucidating the specific surface components of L125 involved in these events, paving the way for targeted interventions at the host-pathogen interface.

## 1 Introduction

Probiotics are live microorganisms that, when administered in sufficient quantities, provide health benefits to the host (Hill et al., 2014). A well-documented feature of probiotic strains is their ability to exert antimicrobial and antibiofilm activity against foodborne and clinically relevant pathogens (Silva et al., 2020). These effects are often attributed to lactic acid production and matrix acidification, and secretion of bacteriocins or other small molecules (Guo et al., 2020). In addition, cell-surface components may exert direct antimicrobial activity (Muscariello et al., 2020) or participate in competitive exclusion events (Tuo et al., 2018). Key components involved in these processes include pilins, adhesins and moonlighting proteins, which mediate adhesion to the ECM and host cell surface, such as fibronectin, collagen, and mucins (Sengupta et al., 2013; Jeffery, 2019). The close proximity of lactobacilli to host cells facilitates trans-kingdom signaling events, influencing critical cellular processes, such as cell death and survival, immune responses, and adhesion to the ECM and neighboring cells (Ye et al., 2023).

*Staphylococcus aureus* and *Escherichia coli* are two common bacteria that can cause localized or invasive infections in hospital and community settings (Ramos et al., 2020; Raineri et al., 2022). Pathogen adhesion and internalization are critical steps during the early stages of infection. *S. aureus* and *E. coli* code for a large repertoire of cell-surface proteins involved in these events, including microbial surface component recognizing adhesive matrix molecules (MSCRAMMs) (Josse et al., 2017), moonlighting adhesins (Furuya and Ikeda, 2011; Harvey et al., 2019), pilins and fimbriae (Croxen and Finlay, 2010). These proteins enable pathogens to attach to mucins and the ECM components, triggering the production of enterotoxins (Younes et al., 2016; Josse et al., 2017). Interactions between pathogens and host proteins such as fibronectin and actin, facilitate their internalization into non-phagocytotic host cells (Sinha and Fraunholz, 2010). This allows pathogens to evade immune surveillance, thus providing a fertile ground for the establishment of chronic infections (Croxen and Finlay, 2010; Foster et al., 2014). Limiting these interactions during the early phases of infection could effectively limit host colonization, and increase pathogen clearance. Therefore, commensals and probiotic microorganisms with the ability to interfere at the pathogen-host interface offer a promising complementary strategy for the prevention or management of life-threatening infections.

In the present study, we examined the ability of two potential probiotic strains, *Lactiplantibacillus pentosus* L33 and *Lactiplantibacillus plantarum* L125, isolated from fermented products (Pavli et al., 2016), to protect HT-29 human colon adenocarcinoma cells against *S. aureus* and *E. coli* infection. As we have previously shown, both strains exerted antimicrobial and antibiofilm effects against these pathogens *in vitro* (Kiousi et al., 2023). In particular, viable bacteria and cell-free culture supernatants (CFCS) reduced pathogen viability in suspension and inhibited biofilm formation (Kiousi et al., 2023). Furthermore, their WGS have been published and are available in public repositories (Stergiou et al., 2021; Tegopoulos et al., 2021). Here, we investigated the ability of L33 and L125 to prevent pathogen cytotoxicity with various experimental setups. *Lacticaseibacillus rhamnosus* GG was used as a reference strain due to its well-documented ability to limit adhesion, biofilm formation, virulence and pathogen-induced cell death *in vitro* and *in vivo*, through secreted metabolites and cell-surface molecules (De Keersmaecker et al., 2006; Spacova et al., 2020). LGG is, also, known to modulate pathogen clearance (Capurso, 2019). Trans-well systems were used to determine whether direct contact between the lactobacilli and host cells is required to prevent pathogen-induced cell death. Subsequently, the ability of the strains to adhere to HT-29 cells and inhibit pathogen adhesion and invasion was evaluated *in vitro*. WGS analysis was performed to identify proteins potentially involved in competitive exclusion events. Finally, dual RNA-seq and protein microarrays were employed to reveal genes and pathways involved in lactobacilli-host interactions and the associated protective phenotype.

## 2 Materials and methods

### 2.1 Bacterial strains and culture conditions

LAB strains L33 and L125 (Pavli et al., 2016) and LGG (DMSZ, Braunschweig, Germany) were cultivated in de Man, Rogosa and Sharp (MRS, Applichem, Darmstadt, Germany) broth at 37°C, under anaerobic conditions. *S. aureus* and *E. coli* were obtained from the microbial collection of the Laboratory of Clinical Microbiology, University Hospital of Alexandroupolis and were routinely cultured in Tryptic Soy Broth (TSB, Condalab, Madrid, Spain) at 37°C under aerobic conditions.

### 2.2 Human cell lines

HT-29 human colorectal adenocarcinoma cell line (ATCC, Manassas, VA, United States) was maintained in Roswell Park Memorial Institute GlutaMAX^™^ (RPMI)-1640 medium supplemented with 10% fetal bovine serum (FBS), 100 μg/mL streptomycin and 100 U/mL penicillin (all from Thermo Fisher Scientific, Waltham, MA, United States). For HT-29-bacteria co-incubations, a modified cell culture medium consisted of 10% FBS and 20 mM 4-(2-hydroxyethyl)-1-piperazineethanesulfonic acid (HEPES; Thermo Fisher Scientific) was used. Cells were incubated in a humidified atmosphere at 37°C, 5% CO_2_ under sterile conditions.

### 2.3 Cell and bacteria viability assays

The cytotoxic capacity of pathogens was initially evaluated using the Sulforhodamine B (SRB) (Invitrogen, Waltham, MA, United States) assay. HT-29 cells were seeded at a density of 7,500 cells per well in a 96-well plate (SPL Life Sciences, Pochon, South Korea) and incubated O/N in standard cell culture conditions. The following day, cells were washed with phosphate-buffered saline (PBS, Thermo Fischer Scientific) and incubated with viable pathogens or conditioned media (CM). For viable cell treatments, pathogens were added at a concentration of 10^7^ CFU/mL for 1, 2 or 4 h. CM was prepared as follows: pathogens were incubated in RPMI-1640 medium supplemented with 10% FBS and 20 mM HEPES for 24 h. Cell pellets were discarded after centrifugation at 8,000 g for 5 min, and the supernatants were sterile filtered using a 0.2 μm filter (Merck, Rahway, NJ, United States). Cells were then treated with pathogen CM for 2 or 4 h. HT-29 cell viability was determined using a previously published protocol (Chondrou et al., 2018). Cell survival (%) is expressed as: [(Sample OD_570_ – Blank OD_570_)/(Control mean OD_570_ – Blank OD_570_)] × 100. Cells treated with standard culture medium served as the untreated control.

The cytotoxic capacity of pathogen CM was also evaluated using the HoloMonitor Live Imaging System (PHI, Boston, MA, United States). In this case, 2 × 10^5^ cells per well were seeded into 24-well plates. The following day, cells were treated with undiluted, sterile-filtered pathogen CM. Cells treated with cell culture medium were included as an untreated control. Cell proliferation was assessed over a 24 h period with the Kinetic Cell Proliferation Assay software (PHI).

The potential protective effects of viable lactobacilli were evaluated against pathogen-induced toxicity, under two different conditions: (i) co-treatment of cells with lactobacilli (10^7^ CFU/mL) and pathogens (10^8^ CFU/mL), (ii) pre-treatment (2 or 4 h) of cells with lactobacilli (10^7^ CFU/mL) prior to the addition of pathogens (10^8^ CFU/mL). Pathogen challenge lasted for 4 or 2 h for *S. aureus* and *E. coli*, respectively. Cell survival was calculated as described above. Untreated cells served as a control, while cells exposed only to pathogens were used as a positive control.

### 2.4 Flow cytometry

Propidium iodide (PI) staining, and flow cytometry were used to examine membrane permeability and cell death. HT-29 cells were seeded at a density of 2 × 10^5^ cells per well in 6-well plates. The following day, cells were pre-treated with lactobacilli (10^7^ CFU/mL) for 4 h after which *S. aureus* (10^8^ CFU/mL for 4 h) or *E. coli* (10^8^ CFU/mL for 2 h) were added. After incubation, cells were collected by trypsinization and were centrifuged at 600 g for 5 min. Then, cell pellets were washed twice with PBS, and stained with 50 μg/mL PI (Biotium, Fremont, CA, United States) for 3 min. Untreated cells served as a negative control, and pathogen-only treated cells as a positive control. Flow cytometry was performed using the Attune NxT Flow Cytometer (Thermo Fischer Scientific) and results were analyzed with FlowJo V10 software (BD Biosciences, San Jose, CA, United States).

### 2.5 Trans-well system

Cell culture inserts were used to determine whether direct contact between L125 and HT-29 cells is required for protection against pathogen-induced cytotoxicity. Specifically, HT-29 cells were seeded at a density of 5 × 10^4^ cells per well into 24-well plates. The following day, L125 cells (10^7^ CFU/mL) were added to either side of a 0.4 μm trans-well insert (SPL Life Sciences) for 4 h. Then, pathogens were inoculated in the lower compartment, in direct contact with HT-29 cells. Cell survival was determined using the SRB assay, after 4 h challenge with *S. aureus* or 2 h challenge with *E. coli*.

### 2.6 Adherence and competitive exclusion assays

HT-29 were seeded in 24-well plates at a density of 4 × 10^5^ cells per well and incubated until reaching 100% confluency, following a previously published protocol (Plessas et al., 2020). Lactobacilli at a concentration of 10^7^ CFU/mL were then added for 2 or 4 h. After incubation, the monolayers were washed twice with sterile PBS, and cells were detached using 1× Trypsin (Thermo Fischer Scientific). The resulting cell suspensions were serially diluted in Ringer’s solution and plated onto MRS agar for enumeration. Plates were incubated at 37°C until visible colonies formed. Attached bacteria are expressed as Log CFU/mL. Lactobacilli-HT-29 interactions were further visualized using an inverted microscope (ZEISS, Jena, Germany).

For the competitive exclusion assay, HT-29 cells were seeded into 24-well plates (4 × 10^5^ cells/well). The following day, cells were pretreated with lactobacilli for 4 h prior to pathogen challenge. Then, pathogens (10^8^ CFU/mL) were added and incubated for 2 h. At the end of the incubation period, the monolayers were washed twice with sterile PBS and cells were disassociated with 1× Trypsin. The suspension was serially diluted in Ringer’s solution and plated onto TSA for colony enumeration of pathogens. The plates were incubated at 37°C, under aerobic conditions, until visible colonies formed. Attached bacteria were expressed as Log CFU/mL. Controls included pathogen-only treated cells and untreated cells to account for potential contamination.

### 2.7 Gentamicin protection assay

HT-29 cells were seeded in 24-well plates at a density of 5 × 10^4^ cells per well. The following day, cells were pre-treated with lactobacilli at a concentration of 10^7^ CFU/mL for 4 h. Pathogens were, then, added at a concentration of 10^8^ CFU/mL for 1 h. Monolayers were washed twice with PBS and incubated in RPMI medium supplemented with 10% FBS and 100 μg/mL gentamicin (all from Thermo Fischer Scientific) for 1 h. Cells were then lysed using 1% (v/v) Triton-X (Thermo Fischer Scientific), the resulting suspension was serially diluted in Ringer’s solution and plated onto TSA plates for colony enumeration of internalized pathogens. Plates were incubated at 37°C, under aerobic conditions, until visible colonies formed. Internalized bacteria were expressed as Log CFU/mL.

### 2.8 Dual RNA-seq

To examine global changes in the expression of genes in L125 and HT-29 cells during co-incubation, dual RNA-seq experiments were performed. Specifically, HT-29 cells were seeded at a density of 8 × 10^5^ cells in 100 mm plates and incubated O/N. The following day, L125 cells were added at a concentration of 10^7^ CFU/mL for 4 h. HT-29 cells treated with cell culture medium were utilized as non-treated control samples. After treatments, plates were washed with PBS, and cells and attached bacteria were collected by trypsinization. Samples were centrifuged at 8,000 g for 5 min and incubated in a lysis buffer containing 1 M Τris-Cl pH 8, 0.5 M EDTA pH 8, 0.1% Triton-X and 100 mg/mL lysozyme, at 37°C for 30 min to maximize the efficacy of RNA isolation from the Gram-positive bacterium L125. Trizol reagent (Sigma-Aldrich, Saint Louis, MO, United States) was finally added, and RNA isolation was performed following manufacturer’s instructions. RNA quality was determined via agarose gel electrophoresis and spectrophotometrically (Thermo Fisher Scientific, NanoDrop 1000 Spectrophotometer). RNA concentration was measured with Qubit according to the Qubit^™^ RNA Broad Range protocol (Thermo Fisher Scientific). Dual RNA-seq (metatranscriptomics) was performed with Illumina NovaSeq X Plus (Strategy PE150). Bioinformatic analysis was performed on the Galaxy server (Abueg et al., 2024). The quality of the obtained reads was determined with FASTQC (v0.74; Andrews, 2010), and low-quality reads were discarded with Trimmomatic (v0.39; Bolger et al., 2014). HISAT2 (v2.2.1; Kim et al., 2019) was used for read assembly against the L125 or human (GRCh38/hg38) genomes. Gene counts and lengths were determined with featureCounts (v2.0.3; Liao et al., 2014) and differential expression with Limma (v3.25.1; Ritchie et al., 2015) and DESeq2 (v1.1.0; Love et al., 2014). Goseq (v1.50.0; Young et al., 2010) and EGSEA (v1.20.0; Alhamdoosh et al., 2017) were used to classify the genes into KEGG pathways and functional categories and for gene ontology analysis, respectively. Transcripts per million (TPM) were calculated for L125 transcripts with StringTie (v2.1.5; Pertea et al., 2015). Based on TPM values expression was characterized as high (>1,000 TPM), medium (10 < TPM < 1,000), low (0.5 < TPM < 10) or below cut-off (TPM <0.5).^1^

### 2.9 *In silico* analysis

The WGS of L125 (Accession number: JAKJPP000000000.1) was searched for S-layer proteins (SLAPS) and proteins containing cell-surface associated domains (LysM, SH3, WxL) and motifs (LPxTG and LPxTG-like) using EggNOG (Huerta-Cepas et al., 2019) and InterPro (Blum et al., 2021). Proteins containing signals for translocation to the bacterial cell surface were predicted using SignalP 6.0 (Teufel et al., 2022). Their localization and topology were also validated with PSORTb (v3.0; Yu et al., 2010) and DeepTMHMM (Hallgren et al., 2022), respectively. Putative surface proteins were selected for further analysis based on the presence of motifs and domains implicated in interactions with host cells and ECM, including mucin-binding domains (MuBPs), fibronectin-binding domains (FnBPs), collagen-binding domains (CnBPs) or proteins containing Ig-like folds and leucine-rich repeats (LRRs). Furthermore, known moonlighting proteins (Jeffery, 2019) were also included. Sequence homology between the annotated cell surface proteins and proteins encoded by *S. aureus* and *E. coli* was examined using Blastp (Camacho et al., 2009).

### 2.10 Docking study

The structure of the putative cell-surface associated proteins containing adhesin-related domains and motifs was predicted with ColabFold (Mirdita et al., 2022). Protein structures of human receptors and proteins participating in the interactions were downloaded from PDB or AlphaFold and used for docking experiments. These include: human fibrinogen chain A (FnA, 1BBR), fibrinogen fragment D (FnB, 1FZA), fibrinogen chain G (FnG, 1DUG), mucin 1 (MUC1, 1SM3), mucin 3b (MUC3b, AF-Q9H195), mucin 4 (MUC4, AF-Q99102), mucin 7 (MUC7, AF-Q8TAX7), mucin 13 (MUC13, AF-Q9H3R2), mucin 16 (MUC16, 7SA9), Toll-like receptor 2 (TLR2, AF-O606063), Toll-like receptor 4 (TLR4, AF-O00206), Toll-like receptor 5 (TLR5, AF-O60602), Toll-like receptor 6 (TLR6, AF-Q6Y2C9), carcinoembryonic antigen-related cell adhesion molecule 1 (CEACAM1, AF-P13688), carcinoembryonic antigen-related cell adhesion molecule 7 (CEACAM7, Q14002), cadherin 1 (CDH1, AF-P12830) and collagen alpha-1(I) chain (COL1A1, AF-P02452). Docking experiments were performed on the HDOCK server (Yan et al., 2020).

### 2.11 Human cytokine array

High throughput determination of cytokines produced by HT-29 cells after co-incubation with L125 was performed using the Proteome Profiler Human Cytokine Array Kit (R&D Systems, Minneapolis, MN, United States). HT-29 cells were treated with the LAB strain at a concentration of 10^7^ CFU/mL for 4 h. Then, culture supernatants were collected, centrifuged at 11,000 g for 5 min and stored at −80°C until further analysis. Supernatants from three independent experiments were pooled and analyzed using the protein microarray, following manufacturer’s instructions. Briefly, the membranes spotted with antibodies against 36 different cytokines, chemokines and acute phase proteins were blocked for 1 h while cell culture supernatants were mixed and incubated with the detection antibody cocktail for 1 h at RT. Then, the membranes were incubated with the sample/antibody mixtures overnight at 4°C on a rocking platform. The following day, membranes were washed three times, incubated with Streptavidin-HRP for 30 min at RT and washed again three times prior to chemiluminescence detection. Membranes were imaged using the ChemiDoc Imaging System (Bio-Rad, Hercules, CA, United States). Mean pixel density was calculated using a protein array analyzer plugin for ImageJ (National Institute of Health, United States).

### 2.12 Statistical analysis

For the statistical analysis of the experimental data, Student’s *t*-test was performed using GraphPad PRISM 9 (GraphPad Software Inc., CA, United States). All experiments were performed in triplicate unless otherwise stated. Results are represented as mean ± standard deviation. A *p*-value <0.05 was considered statistically significant.

## 3 Results

### 3.1 *Staphylococcus aureus* and *Escherichia coli* induce cell death of HT-29 cells in a time-dependent manner

**Figure 1:**
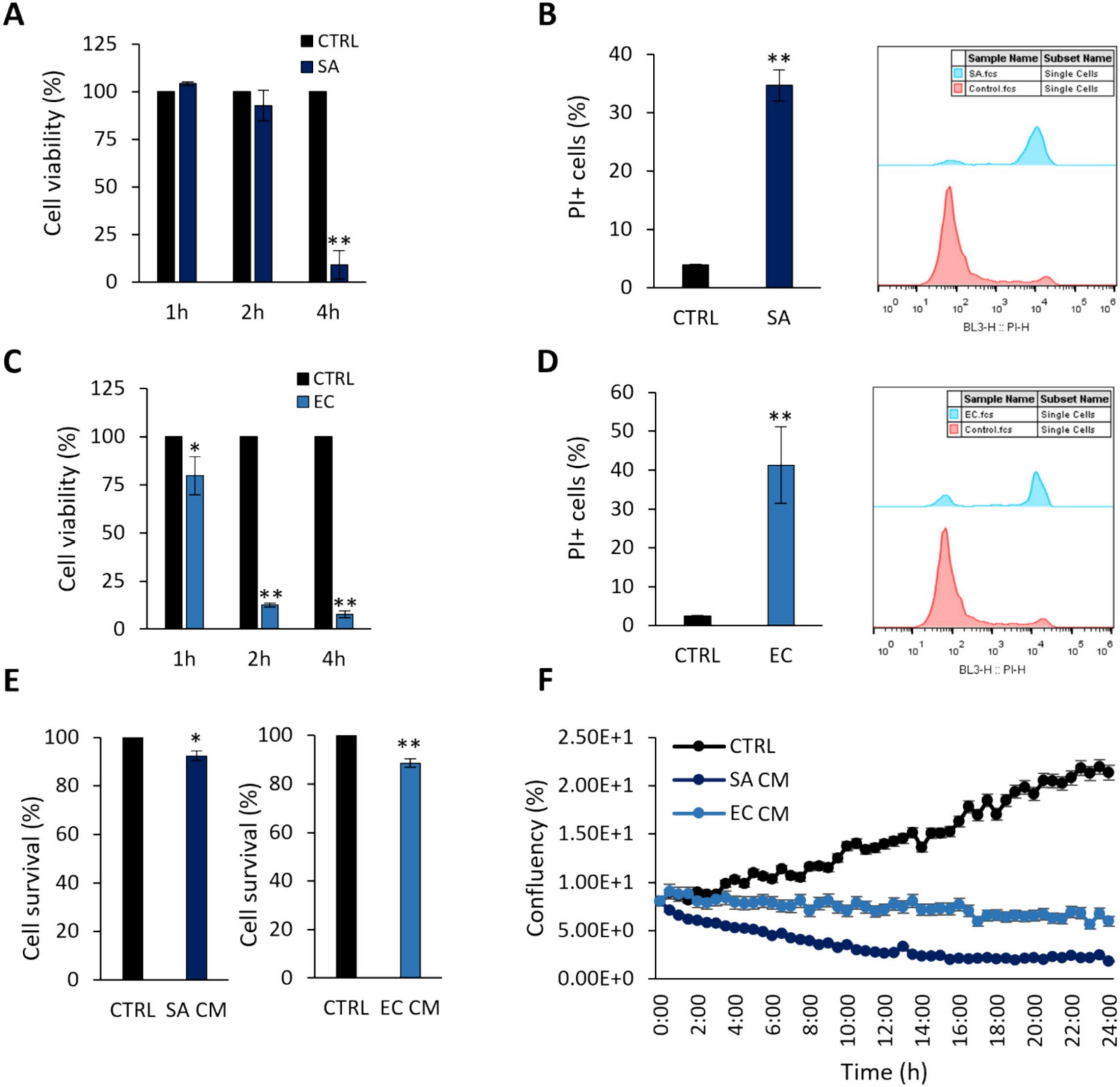
Cytotoxic effects of *S. aureus* and *E. coli* against HT-29 cells. Co-incubation of HT-29 cells with 10^8^ CFU/mL **(A,B)** *S. aureus* (SA) or **(C,D)** *E. coli* (EC) for 1, 2 or 4 h, limited cell viability as measured by **(A,C)** the SRB assay, **(B,D)** PI staining and flow cytometry. **(E)** *S. aureus-*derived CM (SA CM), and *E. coli-*derived CM (EC CM) limited cell viability after 4 h or 2 h of co-incubation, respectively. **(F)** The effect of pathogen-derived CM was monitored for 24 h using the HoloMonitor live cell imaging system. SA CM and EC CM decreased confluency in HT-29 cells in a time-dependent manner. The data presented are the mean ± standard deviation of three independent experiments. ^*^p < 0.05 and ^**^p < 0.005 compared to control (CTRL) untreated cells.

First, we sought to determine the virulence effects of clinically isolated *S. aureus* and *E. coli* strains by their ability to induce cytotoxic effects on the colon adenocarcinoma cell line HT-29 at 1, 2 and 4 h (Figure 1). As shown in Figures 1A,C, a time-dependent reduction in cell viability was recorded for both pathogens. After 4 h-treatment of HT-29 cells with *S. aureus* or 2 h-treatment with *E. coli*, cell viability was reduced by >80% (*p* < 0.005). These timepoints were selected for subsequent experiments. These findings were further validated by PI staining and flow cytometry (Figures 1B,D). The effect of pathogen-derived CM on cell viability after 4 h (*S. aureus*, SACM) or 2 h (*E. coli*, EC CM), was initially investigated with the SRB cytotoxicity assay. A smaller reduction (*p* < 0.005) in cell survival was recorded (Figure 1E), compared to treatments with viable pathogens. These effects were further monitored for 24 h with the HoloMonitor live cell imaging system, using the Kinetic Cell Proliferation Assay software. It was shown that CM derived from either pathogen reduced cell confluency in a time dependent manner (Figure 1F).

### 3.2 L125 pretreatment protects cells from pathogen-induced cytotoxicity

**Figure 2:**
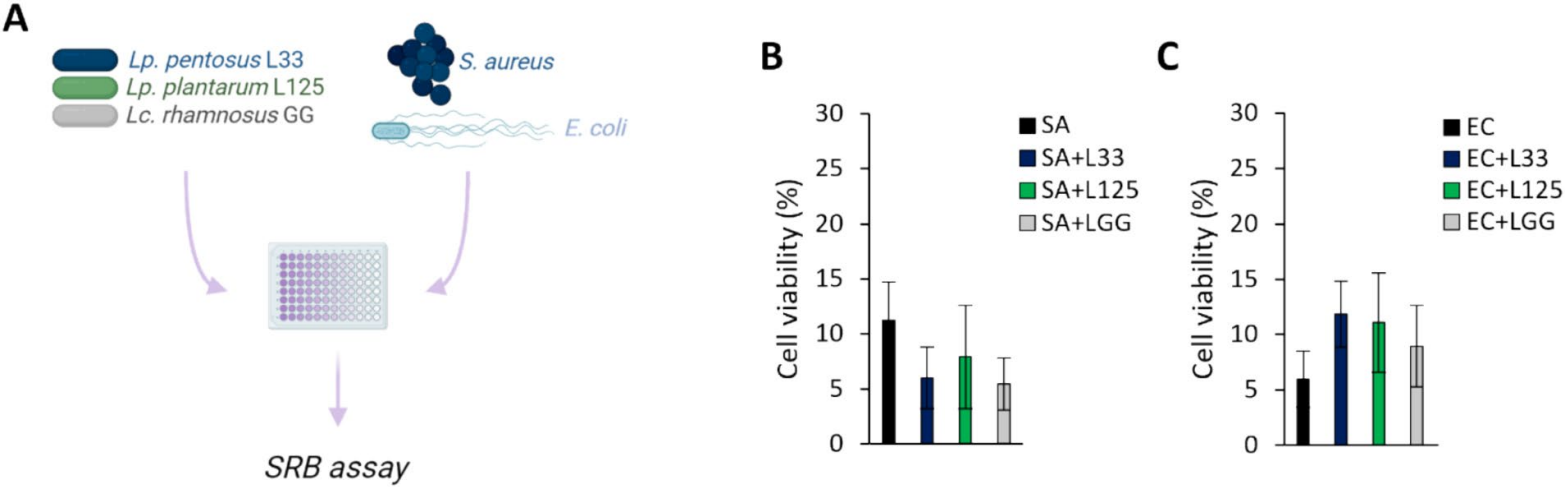
Investigation of the protective effects of viable lactobacilli against pathogen-induced cytotoxicity in co-incubation experiments. **(A)** Schematic representation of the experimental design. Cells were simultaneously treated with 10^7^ CFU/mL of lactobacilli and 10^8^ CFU/mL of **(B)** *S. aureus* (SA) or **(C)** *E. coli* (EC) and cell viability was determined after 4 or 2 h, respectively, with the SRB assay. The data presented are the mean ± standard deviation of three independent experiments.

**Figure 3:**
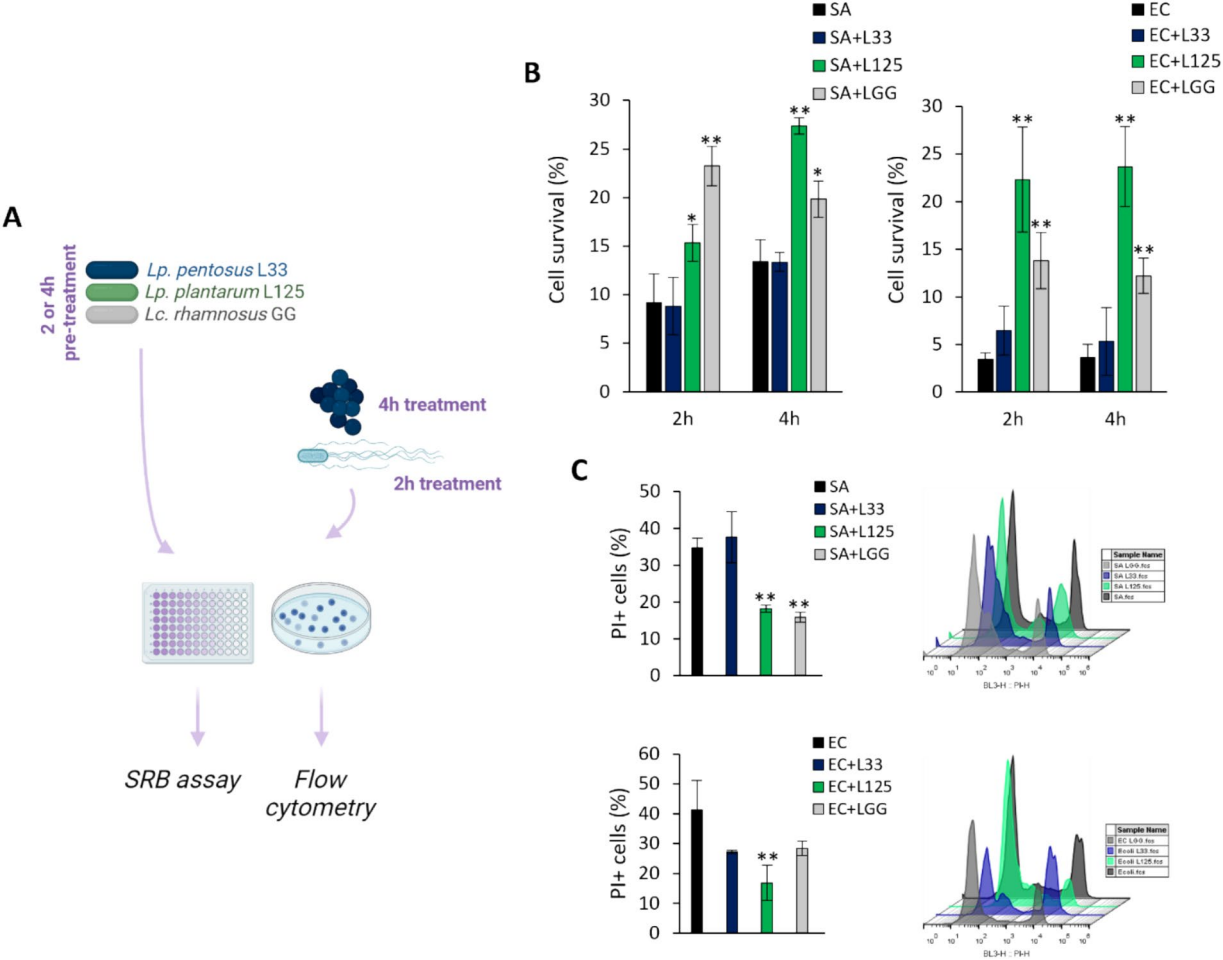
Investigation of the protective effects of viable lactobacilli against pathogen-induced cytotoxicity. **(A)** Schematic representation of the experimental design. **(B)** HT-29 cells were treated with lactobacilli at a concentration of 10^7^ CFU/mL for 2 or 4 h before the addition of the pathogens (10^8^ CFU/mL) (pretreatment). At the end of the incubation period, cell viability was determined using the SRB assay. **(C)** Determination of the protective effect of 4 h-pretreatment of the HT-29 cells against *S. aureus* (SA) or *E. coli* (EC) cytotoxicity via PI staining and flow cytometry. The data presented are the mean ± standard deviation of three independent experiments. ^*^p < 0.05 and ^**^p < 0.005 compared to control infected cells (SA or EC).

The ability of the potential probiotic LAB strains to limit pathogen-induced cell death was then assessed using various experimental designs (Figures 2A,3A). Initially, cells were co-incubated with lactobacilli and pathogens for 4 h (*S. aureus-*treated cells) or 2 h (*E. coli-*treated cells), and viability was determined with the SRB assay. No difference in cell survival (%) was observed between pathogen-only treated cells and those treated with any of the three lactobacilli (Figures 2B,C). Then, we investigated whether lactobacilli pretreatment could prevent pathogen-induced cell death (Figure 3A). As shown in Figure 3B, L125 and LGG significantly limited cell death, while L33 had no significant effect. These results were also confirmed through flow cytometry analysis (Figure 3C). It should be noted that lactobacilli treatment did not affect cell survival (Supplementary Figure S1).

### 3.3 Direct contact of L125 with HT-29 cells confers protection against pathogen-induced cell death

**Figure 4:**
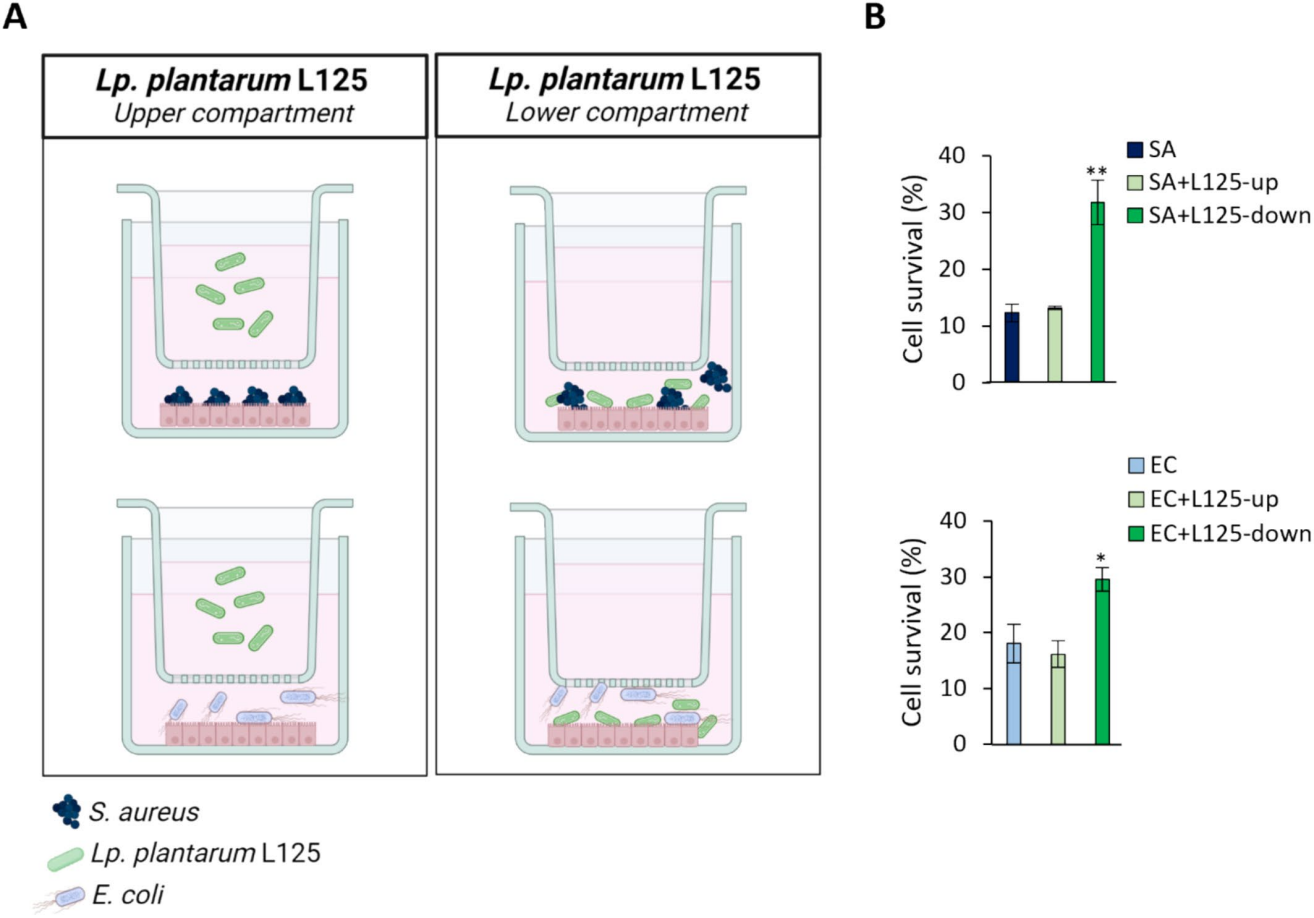
Use of a trans-well cell culture system to determine whether metabolites or cell surface molecules are responsible for the protective effects exerted by L125 against pathogen-induced cytotoxicity. **(A)** HT-29 cells at a density of 5 × 10^4^ cells/well were seeded into the 24-well plates. The next day, L125 was added in the upper (separated) or lower (in contact with the cells) compartment for 4 h. Then, pathogens were added in the lower compartment for 4 (*S. aureus*, SA) or 2 h (*E. coli*, EC). **(B)** Cell viability was determined at the end of the incubation period using the SRB assay. The data presented are the mean ± standard deviation of three independent experiments. ^*^p < 0.05 and ^**^p < 0.005 compared to control infected cells (SA or EC).

A trans-well cell culture system was employed to determine whether direct contact of L125 with the epithelial cells is necessary for protection against pathogen-induced cytotoxicity (Figure 4A). Indeed, direct contact for 4 h prior to the addition of the pathogens resulted in a 2-fold reduction in cell death (*p* < 0.05). However, pretreatment with L125 in the upper compartment of the trans-well system, where no direct contact occurred, did not confer any protective effect (Figure 4B).

### 3.4 L125 limits pathogen adhesion and invasion in HT-29 cells

**Figure 5:**
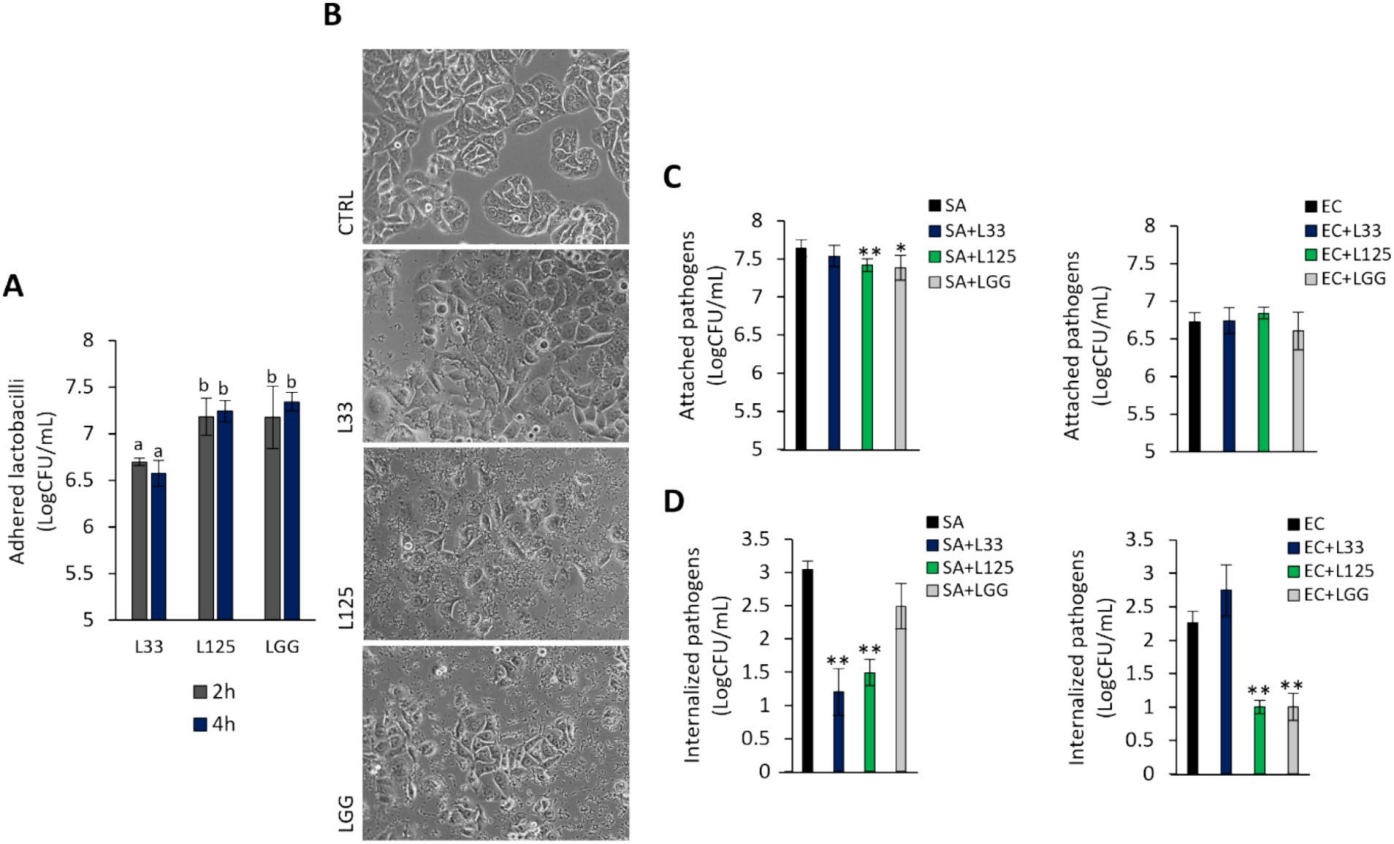
Investigation of lactobacilli-HT-29-pathogen interactions. **(A)** Cells were treated with cell culture medium (CTRL) or lactobacilli at a concentration of 10^7^ CFU/mL for 2 or 4 h. Attached bacteria are expressed as Log CFU/mL. (a, b) Significance was not achieved for variables with the same letter (*p* > 0.05). **(B)** Visualization of the lactobacilli-HT-29 interactions using an inverted optical microscope (ZEISS). **(C)** Competitive exclusion, and **(D)** inhibition of pathogen invasion was determined in cells pretreated with the lactobacilli for 4 h prior to the addition of pathogens. Then, cells were treated with the pathogens for 2 or 1 h for the competitive exclusion or gentamicin protection assay, respectively. Pathogen counts are expressed as Log CFU/mL. The data presented is the mean ± standard deviation of three independent experiments. ^*^p < 0.05 and ^**^p < 0.005 compared to cells incubated with the pathogens (SA or EC).

To further explore the protective phenotype, we measured the capacity of L125 to adhere to HT-29 cells and limit pathogen attachment and invasion, using established microbiological assays. As shown in Figure 5A, L125 and LGG exhibited strong binding to HT-29 cells, while L33 demonstrated markedly lower adherence capacity (*p* < 0.05). Notably, L125 formed aggregates and adopted a net-like morphology over the HT-29 cells (Figure 5B). Pretreatment with L125 or LGG for 4 h reduced the attachment of *S. aureus* (*p* < 0.05), having no effect against *E. coli* (Figure 5C). L33 did not limit the adhesion ability of either pathogen, but did limit internalized *S. aureus* counts. L125 significantly inhibited the invasion of both pathogens in HT-29 cells, reducing counts by more than 1.5 Log CFU/mL (Figure 5D). A slight reduction in pathogen viability was also recorded at the same timepoints (Supplementary Figure S2).

### 3.5 Lactobacilli-HT-29 cells interactions support the expression of adhesins and moonlighting proteins in L125

**Figure 6:**
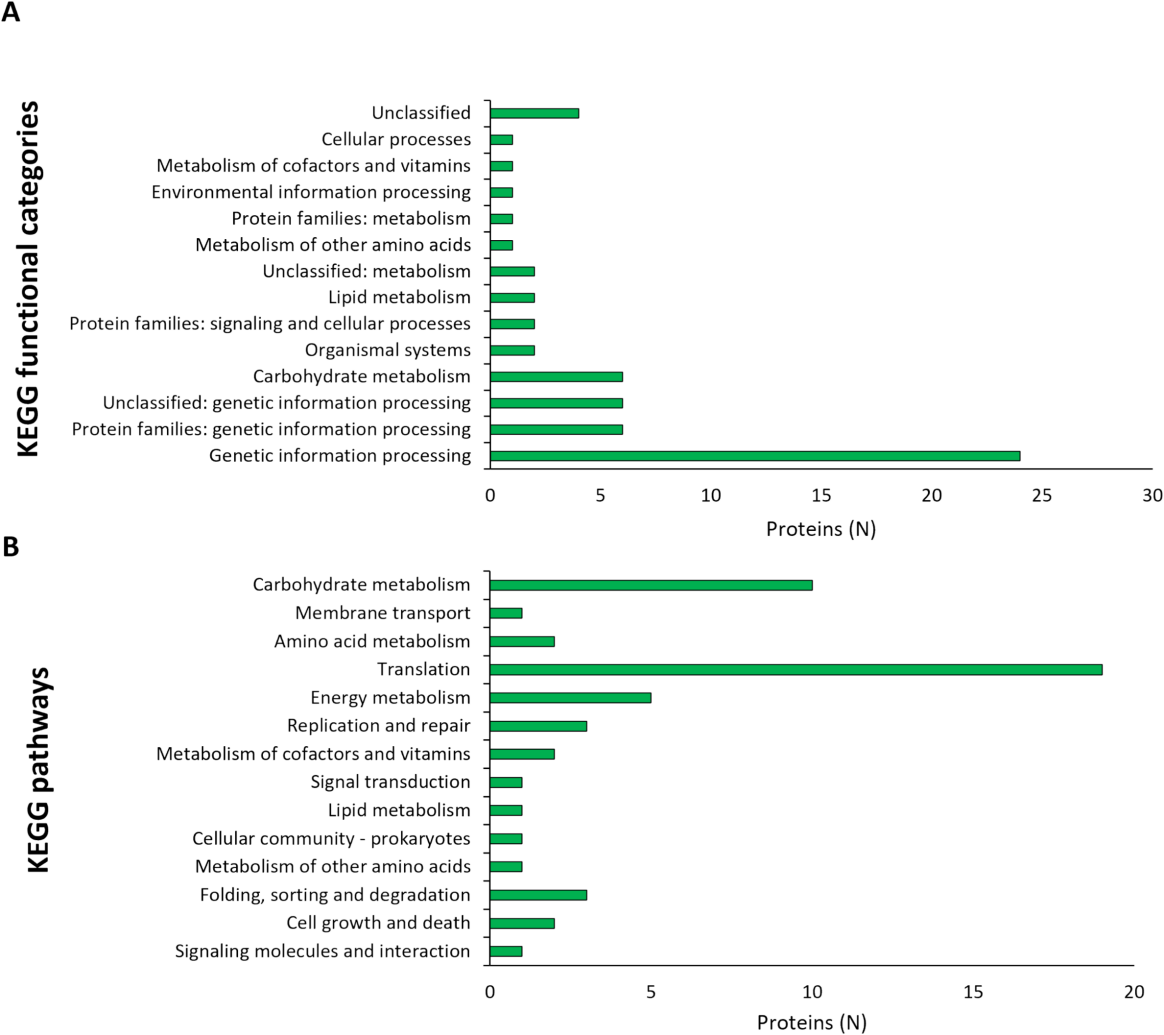
Assignment of L125 transcripts with high expression levels (>1,000 TPM) in **(A)** KEGG functional categories, and **(B)** KEGG pathways using BlastKOALA.

Dual RNA seq was utilized to analyze the gene expression changes in L125 and HT-29 cells during a 4 h co-incubation period. Specifically, for L125, a total of 108 genes exhibited high expression levels (>1,000 TPM), 2.568 genes displayed medium expression levels (10 < TPM < 1,000), and 497 genes showed low expression levels (0.5 < TPM < 10). Additionally, 68 genes were not expressed in neither of the two independent experiments (TPM: 0). The 108 highly expressed genes clustered into 14 KEGG functional categories, 23 KEGG pathways (Figure 6) and 16 clusters of orthologous groups (COGs) (Table 1). The most represented KEGG functional category was “genetic information processing” with “translation” being most prominent KEGG pathway and COG category.

**Table 1:** Classification of L125 highly expressed transcripts (>1,000 TPM) in COG categories.

Category	L125 transcripts
C, Energy production and conversion	4 (4.21%)
D, Cell cycle control and mitosis	1 (1.05%)
E, Amino acid metabolism and transport	1 (1.05%)
F, Nucleotide metabolism and transport	0 (0%)
G, Carbohydrate metabolism and transport	4 (4.21%)
H, Coenzyme metabolism	2 (2.11%)
I, Lipid metabolism	3 (3.16%)
J, Translation	22 (23.16%)
K, Transcription	7 (7.37%)
L, Replication and repair	5 (5.26%)
M, Cell wall/membrane/envelop biogenesis	6 (6.32%)
N, Cell motility	0 (0%)
O, Post-translational modification, protein turnover, chaperone functions	6 (6.32%)
P, Inorganic ion transport and metabolism	4 (4.21%)
Q, Secondary structure	0 (0%)
T, Signal transduction	3 (3.16%)
U, Intracellular trafficking and secretion	1 (1.05%)
V, Defense mechanisms	0 (0%)
S, Function unknown	12 (12.63%)
No category, general function prediction only	14 (14.73%)
Total	95 (100%)

To identify transcriptional changes in cell-surface exposed proteins, the WGS of L125 was, firstly, re-annotated *in silico*. Genome mining was performed to identify proteins potentially involved in the competitive exclusion phenotype, and the strain’s ability to limit pathogen internalization. By filtering the EggNOG output, 77 proteins containing motifs and domains indicative of cell-wall exposure were identified (Supplementary Table S1). Specifically, these proteins included: 17 proteins carrying the WxL domain, 19 proteins with a Gram-positive anchor (i.e., LPxTG or LPxTG-like motifs), 2 proteins with an S-layer homology (SLH) domain and 7 proteins with a SlaP (S-layer assembly protein) domain, 13 proteins containing the LysM domain and 9 proteins with the SH3 domain. Among these proteins: 7 possess Ig-like motifs (Big_2 and Big_3 groups), 5 carry LRR motifs, 3 carry collagen-binding domains (Cna_B), 13 contain mucin-binding domains (MucBP), 2 possess a fibronectin-binding domain (FbpA), 2 carry a FIVAR domain, and 1 has a domain belonging to the concanavalin A-like lectin/glucanases superfamily. Additionally, a protein belonging to the invasin/intimin bacterial adhesion mediator protein family was annotated. Out of these, 29 proteins were predicted to have extracellular localization or be exposed on the cell wall (Table 2), 15 were predicted to be localized to the cytoplasmic membrane, 6 to the cytoplasm, and 31 proteins to have unknown or multiple localization sites. Extracellular and cell wall proteins were selected for further analysis. Most predicted surface proteins were found to utilize a Sec/SPI signal recognized by signal peptidase I (Table 2), and one protein (LP125_RS11350) carries a YSIRK processing signal for transport outside the cell. Finally, 16 moonlighting proteins with adhesin function were identified in the L125 genome (Table 3).

**Table 2:** Putative cell-surface proteins encoded by L125 and their expression levels (TPM) during co-incubation with HT-29 cells.

Locus tag (L125_)	Protein annotation	Localization	Length (aa)	MW (Da)	Signal peptide	Extracellular regions	Putatitive adhesion substrate	TPM
RS10075	SpaA isopeptide-forming pilin-related protein	Cell wall	953	100093.27	SP (Sec/SPI)	29–953	Fibronectin, laminin, collagen, CEACAMs	15.44
RS10525	Peptide ABC transporter substrate-binding protein	Cell wall	547	59905.41	NO_SP	29–547	None	13.45
RS11095	LPXTG cell wall anchor domain-containing protein	Cell wall	1,333	142799.14	SP (Sec/SPI)	36–1,333	Mucins	18.12
RS11130	WxL domain-containing protein	Extracellular	234	23766.4	SP (Sec/SPI)	27–234	None	10.45
RS11335	MBG domain-containing protein	Cell wall	2,043	216373.97	SP (Sec/SPI)	48–2043	Mucins	11.73
RS11350	BspA family leucine-rich repeat surface protein	Extracellular	729	78655.07	Other	13–729	Mucins, LRRs (TLR, FN)	9.57
RS11385	MucBP domain-containing protein	Cell wall	1,176	122749.81	SP (Sec/SPI)	38–1,176	Mucins, LRRs (TLR, FN)	12.82
RS11935	LPXTG cell wall anchor domain-containing protein	Cell wall	504	50693.45	Other	1–504	None	0
RS12380	WxL domain-containing protein	Extracellular	1,135	118669.15	SP (Sec/SPI)	24–1,135	Fibronectin, laminin, collagen, CEACAMs	32.59
RS01150	MucBP domain-containing protein	Cell wall	304	30463.62	Other	1–304	Mucins	1.33
RS01490	SpaA isopeptide-forming pilin-related protein	Cell wall	672	68849.05	SP (Sec/SPI)	29–672	Fibronectin, laminin, collagen, CEACAMs	1.75
RS02875	Bacterial Ig-like domain-containing protein	Cell wall	984	105942.78	SP (Sec/SPI)	28–984	Fibronectin, laminin, collagen, CEACAMs	12.78
RS13800	LamG-like jellyroll fold domain-containing protein	Cell wall	1,093	116475.06	SP (Sec/SPI)	27–1,093	None	8.5
RS02315	CAP domain-containing protein	Cell wall	840	87054.39	SP (Sec/SPI)	29–840	None	25.8
RS02875	Bacterial Ig-like domain-containing protein	Cell wall	1,553	164147.75	Other	1–1,553	Fibronectin, laminin, collagen, CEACAMs	10.83
RS04395	WxL domain-containing protein	Cell wall	1,092	113098.74	SP (Sec/SPI)	32–1,092	Fibronectin, laminin, collagen, CEACAMs	20.64
RS06780	KxYKxGKxW signal peptide domain-containing protein	Cell wall	2,217	236103.07	SP (Sec/SPI)	50–2,217	Mucins	9.29
RS07055	SEC10/PgrA surface exclusion domain-containing protein	Cell wall	903	94272.88	SP (Sec/SPI)	30–903	None	12.42
RS07170	MucBP domain-containing protein	Cell wall	1,257	135631.31	SP (Sec/SPI)	40–1,257	Mucin, fibronectin, laminin, collagen, CEACAMs	15.28
RS08445	WxL domain-containing protein	Extracellular	243	24017.38	SP (Sec/SPI)	29–243	None	0.27
RS09470	LPXTG cell wall anchor domain-containing protein	Cell wall	419	44498.45	SP (Sec/SPI)	28–419	None	17.45
RS09530	Collagen-binding domain-containing protein	Cell wall	811	86877.07	SP (Sec/SPI)	37–811	None	13.26
RS13040	Extracellular protein, NlpC/P60 family, gamma-D-glutamate-meso-diaminopimelate muropeptidase	Extracellular	263	29213.34	Other	1–263	None	211.56
RS01700	SH3 domain-containing protein	Cell wall	785	82115.75	SP (Sec/SPI)	1–785	None	641.4
RS14880	Extracellular protein, gamma-D-glutamate-meso-diaminopimelate muropeptidase	Extracellular	355	35029.95	SP (Sec/SPI)	1–355	None	1661.45
RS05575	N-acetylmuramoyl-L-alanine amidase	Cell wall	282	30955.32	Other	30–282	None	171.39
RS08480	C40 family peptidase	Extracellular	496	48339.32	SP (Sec/SPI)	1–496	None	963.72
RS09840	LysM domain-containing protein	Extracellular	354	35000.44	SP (Sec/SPI)	1–354	None	1171.0

CEACAM, carcinoembryonic antigen-related cell adhesion molecule.

**Table 3:** Moonlighting proteins with adhesin-like functions encoded by L125 and their expression levels (TPMs) during co-incubation with HT-29 cells.

Locus tag (L125_)	Protein annotation	Localization	Length (aa)	MW (Da)	Putatitive adhesion substrate	TPM
RS10155	Maltose phosphorylase mapA1	Cytoplasmic	756	86574.49	ANXA13, PALM	14.95
RS06445	Maltose phosphorylase mapA2	Cytoplasmic	748	85588.49	ANXA13, PALM	1.4
RS02920	Triosephosphate isomerase	Cytoplasmic	252	26971.32	Laminin, fibronectin, integrin	621.45
RS08655	Elongation factor Tu	Cytoplasmic	395	43377.13	Mucin, fibronectin, laminin, actin	8859.1
RS02930	Glyceraldehyde-3-phosphate dehydrogenase	Cytoplasmic	340	36438.13	Mucin, plasminogen	4938.06
RS07515	Elongation factor G	Cytoplasmic	698	76998.67	Fibronectin, plasminogen, laminin, mucins	488.97
RS03220	10 kDa chaperonin	Cytoplasmic	94	10292.69	Mucin	817.33
RS03215	60 kDa chaperonin	Cytoplasmic	541	57437.04	Mucin	1806.17
RS05940	ATP-dependent 6-phosphofructokinase	Cytoplasmic	320	34265.62	Plasminogen	217.86
RS05465	Chaperone protein DnaK	Cytoplasmic	622	66729.7	Fibronectin FN, laminin, collagen	447.11
RS02915	Enolase	Cytoplasmic	442	48029.94	Fibronectin, laminin, plasminogen	954.83
RS01190	Glucose-6-phosphate isomerase	Cytoplasmic	450	49847.97	Laminin, collagen	389.4
RS05000	Glutamine synthetase	Cytoplasmic	448	50972.63	Fibronectin, collagen, laminin, plasminogen	195.69
RS10650	Ornithine carbamoyltransferase, catabolic	Cytoplasmic	340	36854.09	Fibronectin	0
RS02925	Phosphoglycerate kinase	Cytoplasmic	400	42796.86	Plasminogen, actin	486.27
RS06220	Phosphoglycerate mutase GpmB	Cytoplasmic	199	22457.56	Plasminogen	8.8

CEACAM, carcinoembryonic antigen-related cell adhesion molecule.

To investigate possible exclusion events, the homology of L125 surface exposed proteins to those encoded by *S. aureus* or *E. coli* was analyzed *in silico* (Supplementary Tables S2, S3). Several proteins encoded by L125 contain domains with high alignment scores to those found in the two pathogens. Specifically, LP125_RS10075, LP125_RS11095 contain LPXTG anchor domains that show homology to those present in *S. aureus* (HDP6314765.1, HDM8613333.1), LP125_RS11095, LP125_RS11335, LP125_RS01150 and LP125_RS06780 carry MucBP domains that present high similarity to domains encoded by *S. aureus* (HDM8613333.1, EZX22480.1, MDU3924263.1). Furthermore, LRR repeats are contained in proteins encoded by *L. plantarum* L125 (LP125_RS02875, LP125_RS02875) and a BspA family leucine-rich repeat surface protein encoded by *S. aureus* (MDF4035866.1). Accordingly, LRR repeats found in L125 protein LP125_RS11350 share similarity to BspA family leucine-rich repeat surface protein (MBL0960876.1) and a bacterial Ig-like domain-containing protein (WP_142456104.1), respectively. Accordingly, LP125_RS11385, LP125_RS01150, LP125_RS07170 contain MucBP domains with similarity to MucBP domain-containing proteins encoded by *E. coli* (MBC8929979.1, MBC8921713.1). Furthermore, two proteins (LP125_RS01490 and LP125_RS09530) exhibited similarity to collagen-binding proteins (PPI97406.1, HCC5232179.1). The majority of moonlighting proteins annotated in the genome of L125, except for maltose phosphorylase (MapA, LP125_RS10155 and LP125_RS06445), share high sequence similarity with proteins encoded by *S. aureus*. On the contrary, L125 proteins generally exhibit lower query cover and/or similarity with *E. coli* proteins (Table 4).

**Table 4:** Sequence identity of moonlighting proteins encoded by L125 to proteins encoded by *S. aureus* and *E. coli*.

*L. plantarum* L125 locus tag	Hits	Query cover (%)	Identity (%)
RS10155 maltose phosphorylase mapA1	Glycoside hydrolase family 65 protein [*Staphylococcus aureus*]	11	62.64
RS06445 maltose phosphorylase mapA2	Glycoside hydrolase family 65 protein [*Staphylococcus aureus*]	11	67.03
RS02920 triosephosphate isomerase	Triose-phosphate isomerase [*Staphylococcus*]	98	65.2
RS08655 elongation factor Tu	elongation factor Tu [*Staphylococcus aureus*]	99	77.33
RS02930 glyceraldehyde-3-phosphate dehydrogenase gap	Glyceraldehyde-3-phosphate dehydrogenase 1 [*Staphylococcus aureus*]	99	60.06
RS07515 elongation factor G	elongation factor G [*Staphylococcus aureus*]	98	75.22
RS03220 10 kDa chaperonin groS	Co-chaperone GroES [*Staphylococcus aureus*]	96	63.74
RS03215 60 kDa chaperonin groL	Chaperonin [*Staphylococcus aureus* C0673]	97	71.13
RS05940 ATP-dependent 6-phosphofructokinase	6-phosphofructokinase [*Staphylococcus aureus*]	99	65.62
RS05465 chaperone protein DnaK	Molecular chaperone DnaK [*Staphylococcus aureus*]	92	76.31
RS02915 enolase	Phosphopyruvate hydratase [*Staphylococcus aureus*]	97	71.53
RS01190 glucose-6-phosphate isomerase	Glucose-6-phosphate isomerase [*Staphylococcus aureus*]	99	67.19
RS05000 glutamine synthetase	Glutamine synthetase [*Staphylococcus aureus*]	100	68.08
RS10650 ornithine carbamoyltransferase	TPA: ornithine carbamoyltransferase [*Staphylococcus aureus*]	98	64.09
RS02925 phosphoglycerate kinase	Phosphoglycerate kinase [*Staphylococcus aureus*]	100	66.08
RS06220 phosphoglycerate mutase	Hypothetical protein V070_02513 [*Staphylococcus aureus*]	96	50.26
RS10155 maltose phosphorylase mapA1	Glycoside hydrolase family 65 protein [*Escherichia coli*]	95	27.94
RS06445 maltose phosphorylase mapA2	Glycoside hydrolase family 65 protein [*Escherichia coli*]	95	30.74
RS02920 triosephosphate isomerase	Triose-phosphate isomerase [*Escherichia coli*]	100	100
RS08655 elongation factor Tu	Elongation factor Tu [*Escherichia coli*]	100	100
RS02930 glyceraldehyde-3-phosphate dehydrogenase gap	Glyceraldehyde-3-phosphate dehydrogenase [*Escherichia coli*]	99	99.71
RS07515 elongation factor G	Elongation factor G [*Escherichia coli*]	98	63.56
RS03220 10 kDa chaperonin groS	Co-chaperone GroES [*Escherichia coli*]	97	58.70
RS03215 60 kDa chaperonin groL	Chaperonin GroEL [*Escherichia coli*]	97	59.32
RS05940 ATP-dependent 6-phosphofructokinase	ATP-dependent 6-phosphofructokinase [*Escherichia coli*]	94	51.66
RS05465 chaperone protein DnaK	molecular chaperone DnaK [*Escherichia coli*]	98	55.52
RS02915 enolase	Enolase [*Escherichia coli*]	91	99.75
RS01190 glucose-6-phosphate isomerase	Glucose-6-phosphate isomerase [*Escherichia coli*]	31	70.71
RS05000 glutamine synthetase	Type I glutamate—ammonia ligase [*Escherichia coli*]	91	41.53
RS10650 ornithine carbamoyltransferase	Ornithine carbamoyltransferase [*Escherichia coli*]	96	52.12
RS02925 phosphoglycerate kinase	TPA: phosphoglycerate kinase [*Escherichia coli*]	100	42.18
RS06220 phosphoglycerate mutase	Histidine phosphatase family protein [*Escherichia coli*]	69	57.25

Concerning the expression at the transcriptional level, most of the putative cell surface proteins exhibited medium expression levels (100 < TPM < 1,000), with the exception of two extracellular proteins LP125_RS14880 and LP125_RS09840, which had high expression levels (>1,000 TPMs). No transcripts were detected for LP125_RS11935 and very low expression levels were recorded for LP125_RS08445 (TPM < 0.5) (Table 3). As expected, several of the moonlighting adhesins exhibited particularly high expression, reflecting their crucial role in bacterial homeostasis (Table 3). The highest expression levels were recorded for EF-Tu (LP125_RS08655), GAPDH (LP125_RS02930), and Hsp60 (LP125_RS03215). Notably, no transcripts were measured for ornithine carbamoyltransferase (LP125_RS10650).

**Figure 7:**
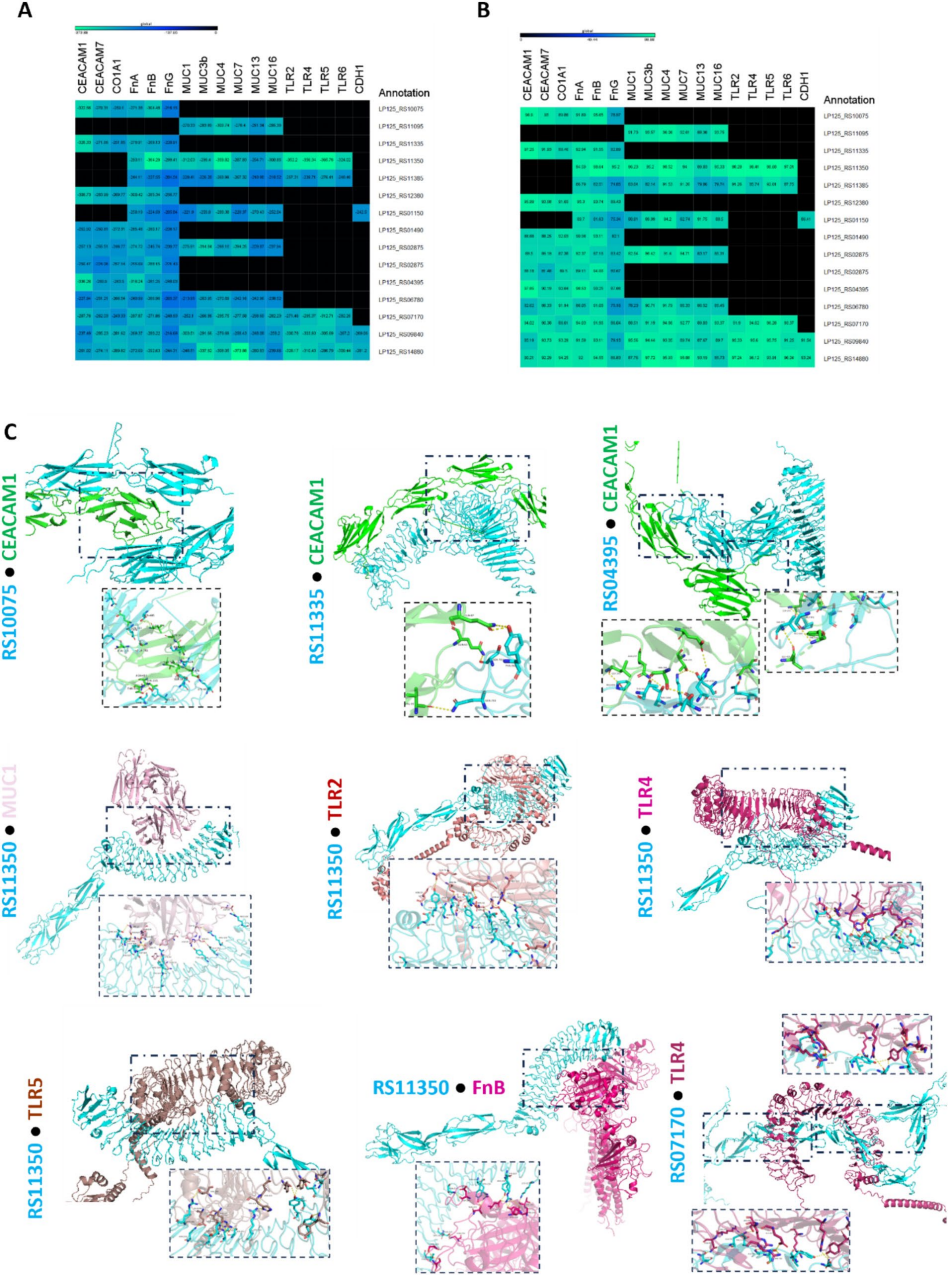
Docking analysis of putative cell surface proteins encoded by L125 with host cell receptors and ECM proteins. **(A)** Docking scores, and **(B)** confidence scores derived from *in silico* docking experiments. **(C)** *De novo* docking of L125 cell surface proteins that present the highest scores with host receptors and ECM proteins. The 3D structure of the annotated proteins was predicted using Collabfold, and host protein structures were derived from PDB or AlphaFold. Docking was performed with HDOCK and the interactions of the proteins presenting the highest docking and confidence were visualized using PyMoL.

**Figure 8:**
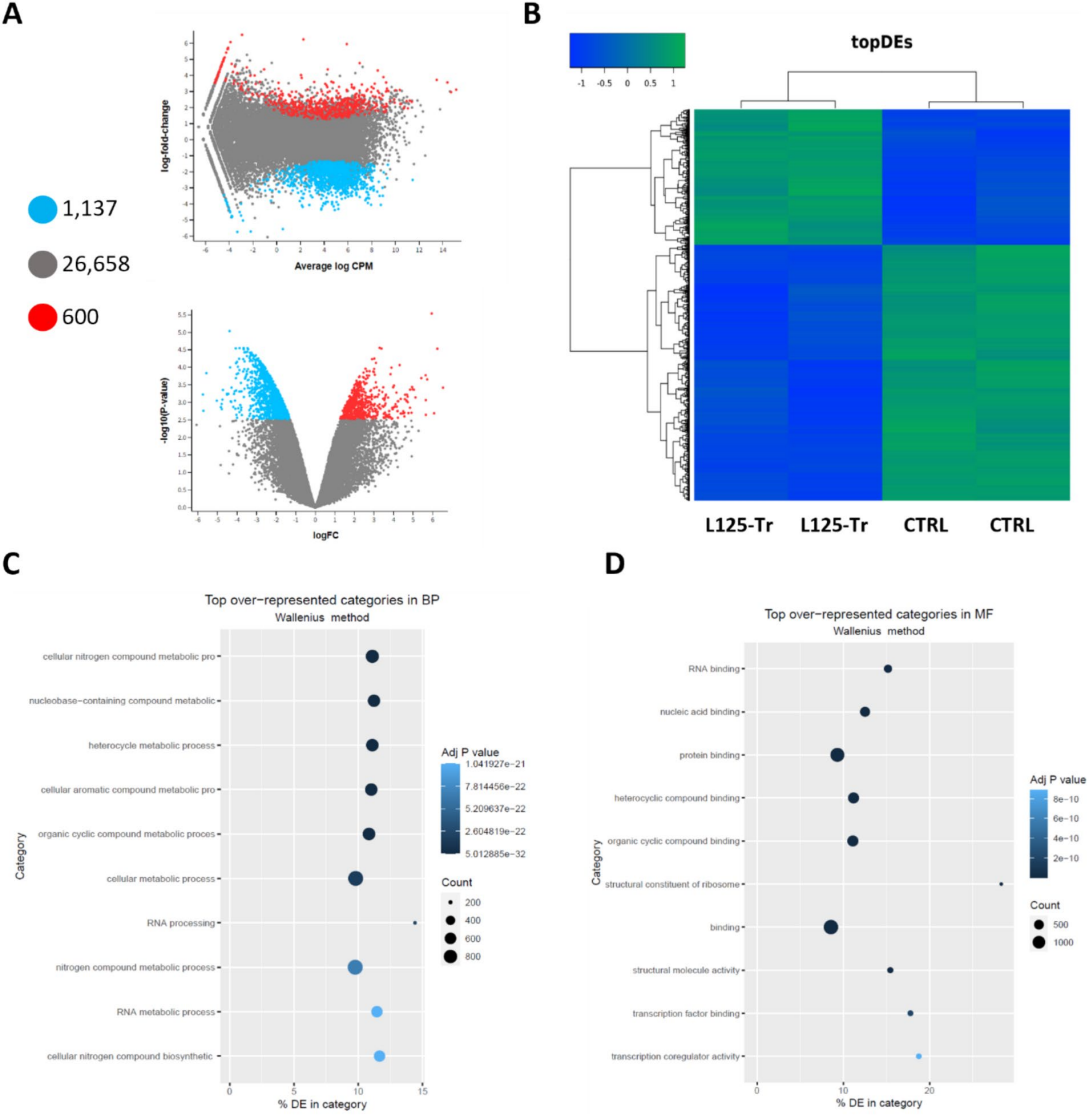
Differential expression analysis in cells treated with L125 for 4 h. **(A)** MD and volcano plots indicating differentially expressed genes in cells treated with L125 (L125-Tr) compared to control, untreated cells (CTRL): 600 genes were upregulated (red dot), 1,137 were significantly downregulated (blue dot), and no change was recorded for 26,658 genes (grey dot). **(B)** Heatmap of the top differentially expressed (topDEs) genes. *Z*-scores were calculated in rows. Top over-represented categories in **(C)** biological processes, and **(D)** molecular function determined with Goseq.

The ability of the annotated, putative cell-exposed proteins to interact with host proteins was determined with *in silico* docking experiments. To this end, docking partners were determined based on the presence of domains and motifs involved in host–microbe interactions (Table 2). As shown in Figures 7, 8 proteins from L125 demonstrated the lowest docking scores (<−300) when interacting with host receptors and ECM components indicating strong interaction potential. More specifically, the LRR or Ig-like domains contained in LP125_RS10075, LP125_RS11335, LP125_RS12380 and LP125_RS04395 interact with CEACAM1 with a high confidence score (>95%). Accordingly, LP125_RS10075 and LP125_RS11350 interact with FnB (confidence score >95%) and LP125_RS12380 with FnA (confidence score 95.3). Furthermore, the MucBP and LRR domains present in LP125_RS11350 interact with MUC1, 4 and MUC16, as well as with LRR domains present in TLR2, 4, 5, 6 with a confidence score >97%. Additionally, the Ig-like folds of LP125_RS07170 demonstrated interaction with MUC4, TLR4 and 5 with a confidence score >94%. Interestingly, the highly expressed LP125_RS14880 and LP125_RS09840 proteins, which do not contain specific adhesin-related domains or motifs, may still contact extracellular domains of TLR4 and −5. Moreover, LP125_RS09840 may also form interactions with MUC1 and LP125_RS14880 with MUC3b and 7, as indicated by the low docking scores and high confidence scores (Figures 7A,B). Protein–protein interactions of partners with the lowest free energy are presented in Figure 7C.

### 3.6 Microbe-host interactions alter gene expression in HT-29 cells

**Figure 9:**
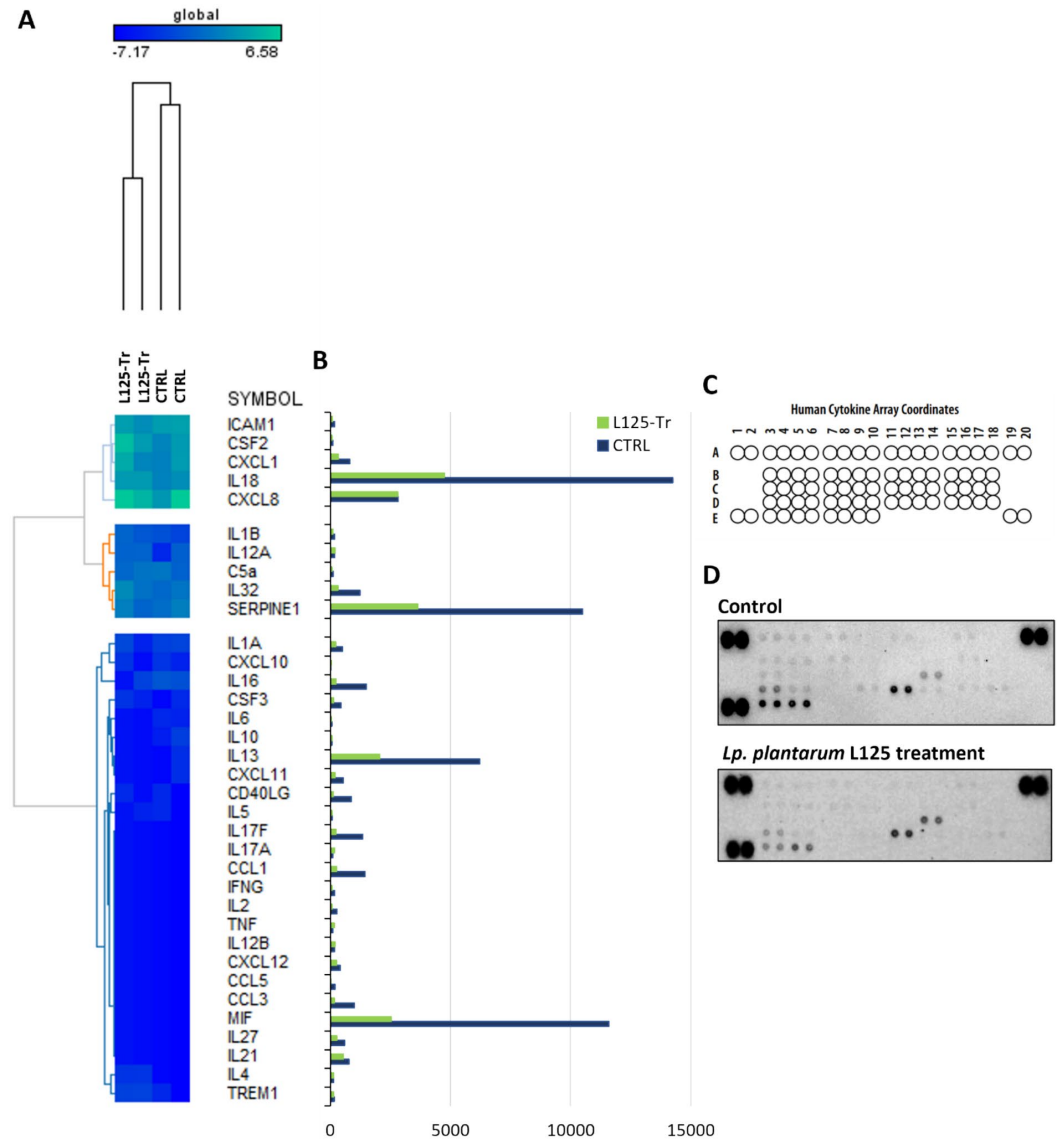
Investigation of the effect of L125 on the expression of immune-related genes in HT-29 cells. **(A)** The expression levels of cytokines and chemokines were determined at the transcriptional level with RNA-seq. Heatmaps of normalized counts of two independent experiments calculated with Limma-voom were constructed on GENE-E. **(B,C)** The effect of lactobacilli treatment (4 h) (L125-Tr) on the expression of 36 immunological markers in HT-29 cells was estimated using the Proteome Profiler Human Cytokine Array Kit. The supernatants of three independent experiments were pooled and utilized in the assay. Untreated cells were used as control (CTRL). **(D)** Mean pixel density of spots was calculated using the protein array a protein array analyzer plugin for ImageJ. Pairs of spots in the upper-left, upper-right and down-left corners are positive controls.

**Figure 10:**
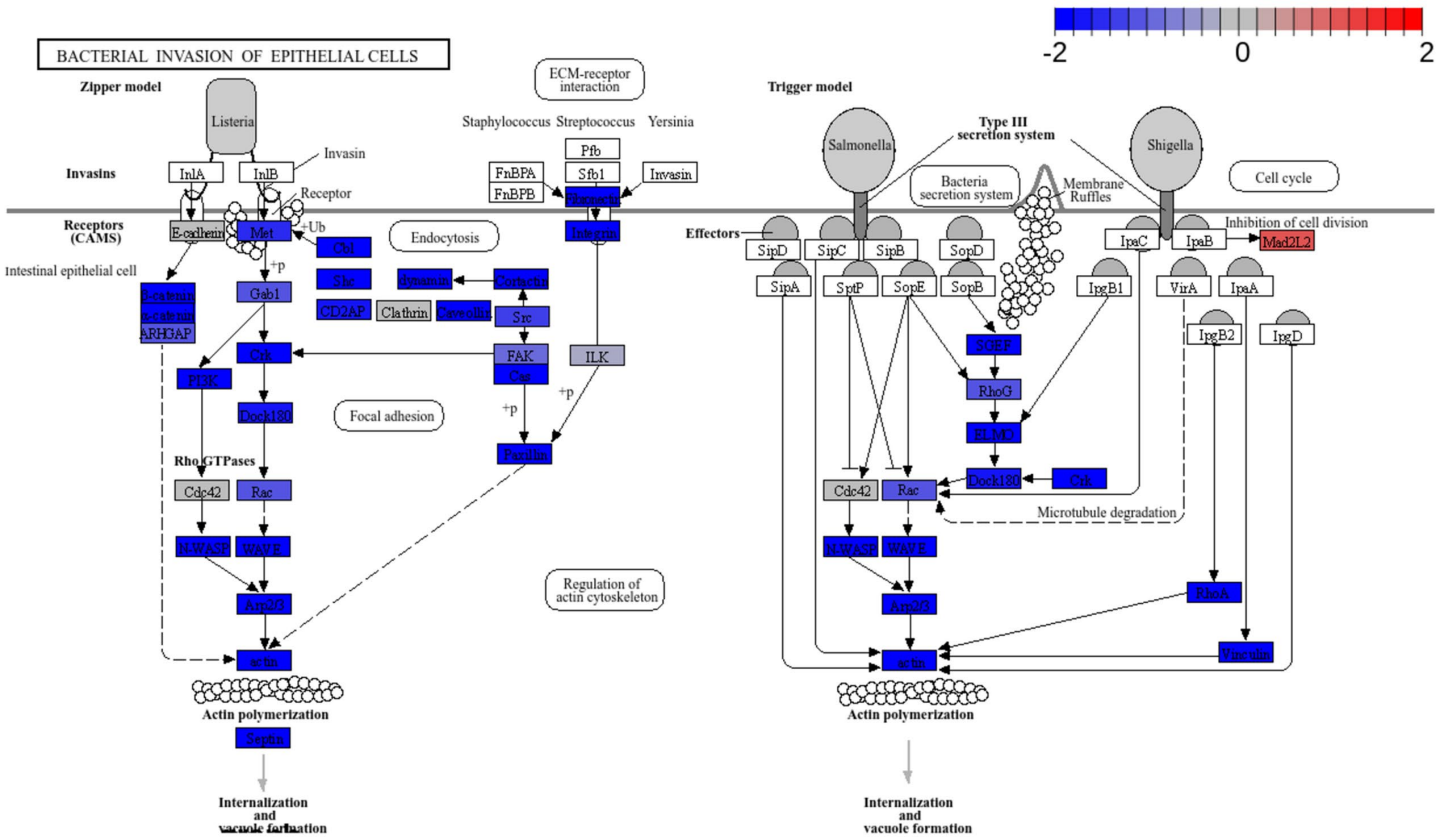
Pathway enrichment analysis of cells treated with *L. plantarum* L125 vs. untreated cells. Pathway “Bacterial invasion of epithelial cells” (hsa05100) was downregulated. Pathway enrichment analysis was performed with EGSEA and the visualization of KEGG pathways with Pathview.

RNA seq was utilized to explore the capacity of L125 to prime antimicrobial responses in the host cell. As shown in Figure 8, treatment of cells with L125 for 4 h led to the upregulation of 600 genes and the downregulation of 1,137 genes in HT-29 cells, while 26,658 genes remained unaffected. The top over-represented categories in “biological function” and in “molecular function” are visualized in Figures 8C,D. Interestingly, L125 did not significantly modulate immune-related pathways, including TLR- or NOD- signaling cascades, nor did it affect the production of cytokines and chemokines (Figure 9A). At the protein level, L125 was shown to limit the secretion of immunological markers in pooled cell culture supernatants, collected from three independent experiments (Figures 9B–D). Pathway analysis revealed that L125 primarily exerted inhibitory effects (Supplementary Table S4), negatively impacting bacterial invasion (Figure 10), adherens junction (Supplementary Figure S3), and endocytosis (Supplementary Figure S4). These phenotypes are mediated by central processes related to the regulation of actin cytoskeleton, internalization and vacuole formation. In this context, genes involved on the mitotic spindle assembly pathway and G2/M progression were also downregulated, further emphasizing L125’s regulatory role in cellular processes.

## 4 Discussion

Probiotic LAB employ various direct and indirect antimicrobial mechanisms to interfere with the host-pathogen interface, thereby limiting pathogen viability, adhesion and biofilm formation. These mechanisms include the production of antimicrobial metabolites and bacteriocins, competitive exclusion and competition for nutrients and resources (Arqués et al., 2015). Concomitantly, certain strains can also modulate cellular signaling to promote pathogen clearance (Llewellyn and Foey, 2017). *S. aureus* and *E. coli* are pathogens of clinical significance, that can invade the epithelial barrier and cause serious systemic and blood-borne infections. The production of membrane-damaging toxins (i.e., hemolysins, leukotoxins, leucocidin) and toxins that interfere with receptor function (i.e., enterotoxins and enterotoxin-like toxins) is upregulated by adhesion and biofilm formation in the host niche, supporting pathogen internalization and dissemination (Younes et al., 2016; Josse et al., 2017). Therefore, limiting these early interactions is a key to preventing the deleterious effects of infection.

We have previously shown that *L. pentosus* L33 and *L. plantarum* L125 possess potent antimicrobial and antibiofilm potential against *S. aureus* and *E. coli* (Kiousi et al., 2023). In this study, we aimed to elucidate the potential of these strains to inhibit infection and prevent loss of cell viability. Based on our findings, L125 was capable of limiting pathogen-induced cytotoxicity in HT-29 cells, when in direct contact for 4 h prior to *S. aureus* or *E. coli* addition. These inhibitory effects induced by L125 were comparable to those observed with LGG, a strain known for its ability to inhibit *S. aureus-*induced cytotoxicity (Spacova et al., 2020; El-Chami et al., 2022). Notably, this is the first study to report that LGG can also prevent *E. coli-*induced cell death in HT-29 cells. In contrast, pretreatment with L33 did not prevent pathogen-induced cell death, but significantly decreased internalized *S. aureus* counts. We previously demonstrated that L33 promotes adhesion of *S. aureus* in co-incubation with HT-29 cells (Kiousi et al., 2023), suggesting that this phenotype could be attributed to steric hindrance or other specific interactions. Additionally, the cytoprotective effects of L125 and LGG were also recorded in the melanoma cell line A375 (data not shown). Similar direct contact requirements have been reported for other potential probiotic strains, such as *L. jensenii* RC-28 or *L. reuteri* RC-14, which limited adhesion, pathogen-induced inflammation and production of *S. aureus* toxin TSST-1 in the vaginal epithelial cell line VK2-E6E7 (Younes et al., 2016). Likewise, *L. fermentum* 8,711 increased the viability of Caco-2 infected with methicillin-resistant *S. aureus* (Jayashree et al., 2018). In this context, we hypothesized that the incubation period allows for the establishment of a protective net that limits pathogen adhesion and invasion and/or primes host responses towards pathogen clearance. To test this hypothesis, we employed *in silico* and *in vitro* approaches.

First, the capacity of L125 to exclude pathogen adhesion and invasion was examined, after a 4 h pretreatment period. A modest, yet significant, reduction in *S. aureus* attachment to HT-29 cells was recorded (~0.2 Log CFU/mL). Νotably, L125 demonstrated a higher capacity to limit invasion of both *S. aureus* and *E. coli* in HT-29 cells by more than 1.5 Log CFU/mL. Subsequently, we sought to investigate potential lactobacilli-host and lactobacilli-pathogen interactions, *in silico*, by re-annotating the genome of L125. The predicted surface proteins of L125 include those anchored on the cell wall through covalent interactions (LPxTG and LPxTG-like motifs) or non-covalent interactions (LysM, SH3 and WXL domains), contain domains involved in adhesion to host components like mucins (MucBPs) and fibronectin (FbpA), as well as interactions with TLRs via LRR domains and CEACAMs through Ig-like domains. Specifically, for *S. aureus*, which showed reduced cell attachment and internalization in HT-29 cells pretreated with L125, these motifs and domains are conserved in MSCRAMMs and utilized during host colonization and epithelial invasion. Previous studies have showed that LGG exerts cytoprotective effects via pilli encoded by the *spaCBA* cluster, which block *S. aureus* attachment to keratinocytes (Spacova et al., 2020). Similarly, moonlighting proteins with adhesin function, enolase, GAPDH, EF-Tu, and TpiA have been found to form transient interactions with host proteins, like fibrinogen, fibronectin, and cytoskeletal proteins (Jeffery, 2019). Among the annotated proteins in this study, only moonlighting adhesins exhibited significant similarity to proteins encoded by the two pathogens, alluding to their central role in mutual exclusion phenomena. In this context, enolase and TpiA encoded by LGG were found to inhibit *S. aureus* binding and cytotoxic effects on keratinocytes, in a dose-dependent manner (El-Chami et al., 2022). In addition to genome analysis, we examined the expression levels of adhesins and moonlighting proteins with adhesin function in L125 during co-incubation with HT-29 cells. As anticipated, moonlighting proteins participating in crucial housekeeping functions were highly expressed. Most adhesins were expressed in medium levels, with the exception of two extracellular proteins (LP125_RS14880, LP125_RS09840), which were expressed at high levels. These findings suggest that during the pre-incubation period these adhesins and moonlighting proteins may help anchor L125 to the HT-29 cell surface, promoting competitive exclusion via specific interactions and steric hindrance. Interestingly, we previously showed that L125 presents high co-aggregation capacity with *S. aureus*, which could further contribute to its ability to limit pathogen adhesion and invasion (Kiousi et al., 2023). These interactions may play a key role in the observed protective effects of L125 in cellular models.

Bacterial cell-surface proteins with adhesion capacity can participate in trans-kingdom signaling events. In this context, lactobacilli are shown to induce strain-specific immunomodulatory effects that are mainly mediated by cell-surface MAMPs or excreted metabolites (Wells, 2011). Several annotated proteins in the genome of L125 were shown to possess LRR domains that can interact with TLRs. TLR-2 activation by *L. plantarum* strains has been linked to strengthening the gut barrier by upregulating and enhancing trafficking of zona occludens-1 (ZO-1) and occludin (Karczewski et al., 2010), while also stimulating pro-inflammatory responses (Fidanza et al., 2021). On the other hand, *L. plantarum* glycolipids were shown to trigger TLR-2/TLR-6 signaling, leading to tolerogenic IL-10 responses and the induction of regulatory T (Treg) populations (Karczewski et al., 2010). MUCs, also, play a role in regulating cellular survival and immune responses. For instance, the formation of the MUC4-ERBB2-ERBB3-NRG1 complex can suppress apoptosis (Carraway et al., 2002), while MUC13 has been identified as an oncogenic glycoprotein that promotes cancer cell growth (Sheng et al., 2016; Pang et al., 2022). Regarding the immunomodulatory capacity of these proteins, MUC1 and MUC16 were found to mask TLR signaling, resulting in the reduction of effector T cell functions, therefore enhancing cancer cell immune evasion and metastasis (Bhatia et al., 2019). Pro-inflammatory cytokines participate in pathogen clearance via the induction of local immune response and modulate the production of antimicrobial peptides and tight junction proteins from the epithelium, contributing to gut homeostasis. Indeed, the IL-1 family cytokines (i.e., IL-1α, IL-1β and IL-18) have been shown to upregulate transcription of defensins, iron-sequester lipocalin, as well as psoriasin and calprotectin (Kolls et al., 2008). Meanwhile, IL-8 and MCP-1 can exhibit direct antimicrobial effects (Yount et al., 2006). Moreover, short-term exposure to IL-10, IL-4, IFN-γ, IL-39γ, IL-6 and IL-22, and low levels of TNF-α can stimulate tissue regeneration and proliferation (Andrews et al., 2018). In this study, dual RNA-seq and protein microarrays were used to study immunomodulatory events that may contribute to pathogen clearance and resistance to pathogen-induced cell death. At the transcriptional level, L125 did not significantly alter cytokine or chemokine levels, but decreased the secretion of specific immunological markers in the cell culture medium. This aligns with our previous findings that immune response induction by potential probiotic lactobacilli in non-professional antigen-presenting cells requires prolonged incubation (Chondrou et al., 2020), suggesting that the short incubation period in this study may not have been sufficient for significant changes in immunological marker levels. Finally, L125 did not affect the production of antimicrobial peptides, as cathelicidin and defensin levels remained unchanged between treated and untreated cells. In the future, metabolomic and proteomic approaches will be used to further characterize the bidirectional L125-host interactions and validate the mechanisms involved in the recorded protective phenotype. In this context, Q Exactive-Based Quantitative Proteomics shown that *Lactobacillus mucosae* strain LM1 could affect tight junction assembly, and cellular and metabolic processes in IPEC-J2 intestinal epithelial cells (Pajarillo et al., 2017). At the same time, similar approaches have been applied to probe the *Salmonella* spp. (Birk et al., 2024), *Pseudomonas auruginosa* (Liu et al., 2024), and *Acinetobacter baumanii* (Li et al., 2021) - host crosstalk, providing significant insight into the early and late stages of infection. The application of proteomics in the context of the lactobacilli-host-pathogen interface can provide novel insight into the capacity of lactobacilli to limit the detrimental effects of infection (Stastna, 2024).

Pathway enrichment analysis revealed a significant downregulation of key signaling pathways involved in endocytosis, adherens junction formation, and the attachment and invasion of epithelial cells by pathogens. To the best of our knowledge, this is the first study to use dual RNA-seq to investigate the priming effects of lactobacilli on cellular function, in co-culture experiments. The affected modules are predominantly exploited by pathogens that colonize and internalize into host epithelia. In this context, *S. aureus* utilizes Fn-binding proteins to engage integrin α5β1, re-organize the actin cytoskeleton and generate focal contacts by regulating protein tyrosine kinase signaling and focal adhesion kinase activity (Hauck and Ohlsen, 2006). Similar integrin-mediated interactions that result in internalization are also utilized by *Streptococcus* spp. (Wang et al., 2007), *Yersinia* spp. (Matsumoto and Young, 2009), and certain *E. coli* pathotypes (Pokharel et al., 2023). Additionally, *E. coli* employs proteins with Ig domains to interact with host cell surface molecules like CEACAMs (van Sorge et al., 2021). Indeed, CEACAM-1 and -6 binding is used by pathogens to invade target cells (Pakbin et al., 2021). Several lactobacilli strains have been shown to reduce pathogen internalization, including *L. salivarius* W24, *L. rhamnosus* W71 (Campana et al., 2017), *L. gasseri* LG-7528, *L. crispatus* LCR-A21, *L. paracasei* LPP-A16 and *L. rhamnosus* LR-B5 (Poimenidou et al., 2023), and *L. helveticus* R0052 (Wine et al., 2009), in studies utilizing the gentamicin protection assay. Curiously, L125 upregulated the production Mad2L2, a protein involved in cell cycle arrest, an effect previously reported for *Shigella*, which exploits the epithelial renewal mechanisms to promote perseverance in the intestinal niche (Iwai et al., 2007).

**Figure 11:**
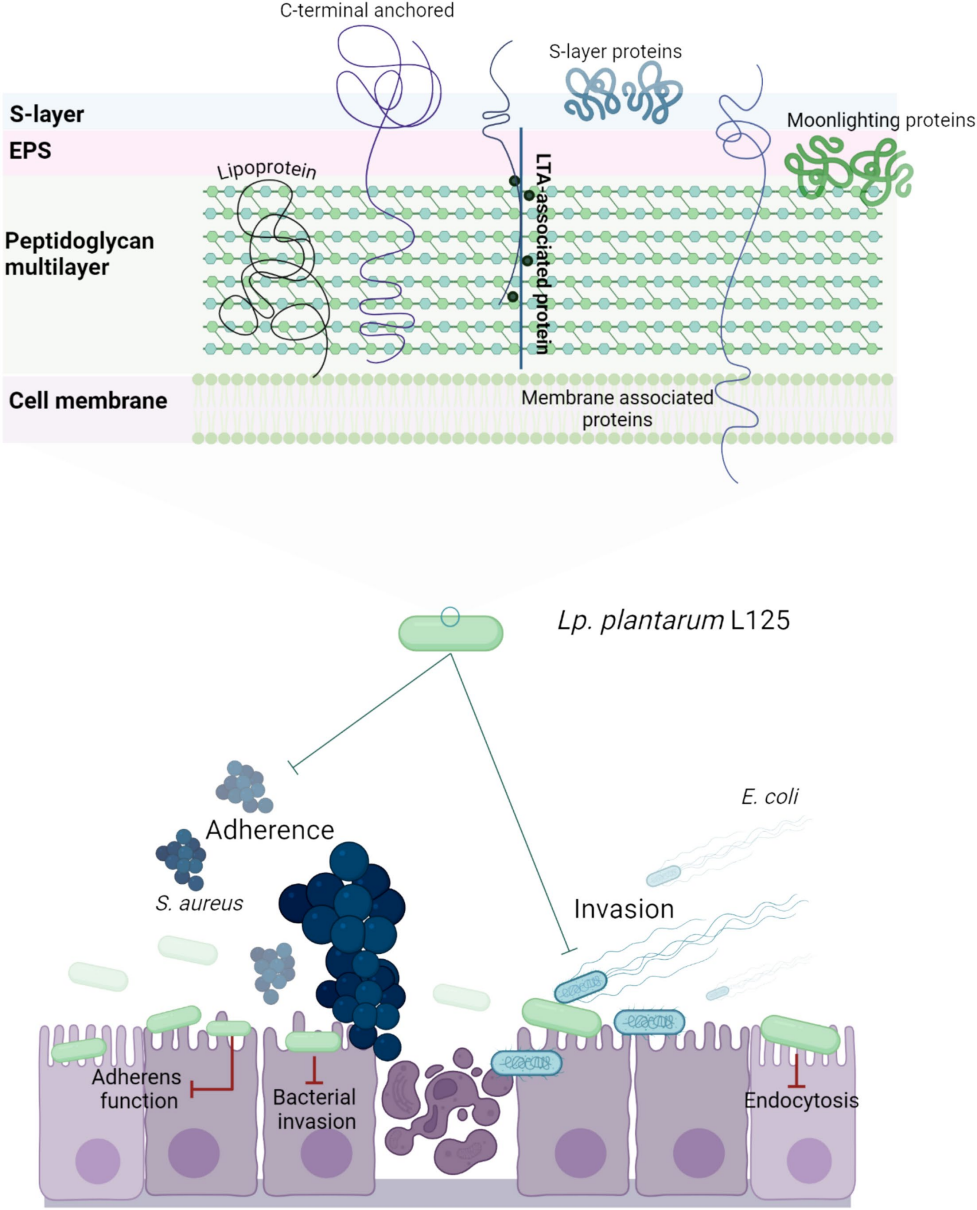
Schematic representation of a putative mechanism by which *L. plantarum* L125 prevents pathogen-induced cell death. L125 cell surface is decorated by C-terminal anchored proteins containing LPxTG, WxL, SH3 or LysM motifs, S-layer proteins carrying the SLH domain, and moonlighting proteins with adhesin function. Adhesins form covalent bonds with cell wall or membrane components, while moonlighting proteins form reversible interactions based on their charge and hydrophobicity. L125 pretreatments resulted in the formation of a protective layer on epithelial cells, preventing the invasion of *S. aureus* and *E. coli*. At the same time, L125 downregulated pathways involved in pathogen adhesion and internalization, endocytosis, cell–cell adherence, and actin cytoskeleton formation.

Overall, our findings highlight the strain-specific ability of lactobacilli to enhance epithelial resistance against pathogenic insults via interference with host-pathogen interactions and regulation of pathogen internalization pathways (Figure 11). Future studies will focus on the identification of the specific surface components of *L. plantarum* L125 that orchestrate these events. Understanding these novel interactions may provide deeper insights into microbe-host interactions under homeostatic conditions and offer new strategies for targeting the host-pathogen interface.

## 5 Conclusion

Probiotics possess several mechanisms to limit pathogen viability and infectivity. In this study, the cytoprotective effects of two potential probiotic LAB strains against *S. aureus* and *E. coli-*induced cytotoxicity were examined using *in vitro* and *in silico* approaches. Among the strains tested, L125 exhibited a strong ability to limit cell death via direct contact with HT-29 cells for 4 h prior to pathogen exposure. Concomitantly, L125 significantly reduced pathogen internalization (>1.5 log reduction) and limited *S. aureus* attachment. Genome annotation of L125 revealed cell surface-associated and extracellular proteins likely involved in competitive exclusion events. Finally, microbe-host interactions triggered the expression of proteins with adhesin function in L125, and downregulated pathways related to endocytosis, adherence and pathogen internalization in epithelial cells. Future studies will focus on the identification and characterization of bacterial components that mediate the recorded cytoprotective effects.

## Data Availability

The datasets presented in this study can be found in online repositories. The names of the repository/repositories and accession number(s) can be found at: https://www.ncbi.nlm.nih.gov/, JAIGOE000000000.1; https://www.ncbi.nlm.nih.gov/, PRJNA1162724.

## Footnotes

^1^EMBL-EBI, Expression Atlas. Available on: https://www.ebi.ac.uk/gxa/FAQ.html.
