# Supplementary material for: Lactobacilli-host interactions inhibit Staphylococcus aureus and Escherichia coli-induced cell death and invasion in a cellular model of infection
Source: Front Microbiol. 2024 Dec 18;15:1501119. doi: 10.3389/fmicb.2024.1501119 (PMC11688250; doi:10.3389/fmicb.2024.1501119)
Supplement: Supplementary file 2 [file Table_2.DOCX]

**Supplementary Table 2.** Sequence identity of cell-surface proteins encoded by L125 to proteins encoded by *S. aureus.*

| ***Lp. plantarum L125* locus tag** | **Blastp Hit** | **Max score** | **Total score** | **Query cover** | **E-value** | **Identity (%)** | **Positive substitutions (%)** | **Gaps (%)** | **Accesion** |
| --- | --- | --- | --- | --- | --- | --- | --- | --- | --- |
| LP125_RS10075 | TPA: LPXTG cell wall anchor domain-containing protein [Staphylococcus aureus] | 114 | 114 | 16% | 2.00E-24 | 37.65% | 55.00% | 21.00% | HDP6314765.1 |
| LP125_RS10525 | peptide ABC transporter substrate-binding protein [Staphylococcus aureus] | 378 | 378 | 92% | 2.00E-123 | 37.50% | 58.00% | 0.00% | MDF4035157.1 |
| LP125_RS11095 | TPA: LPXTG cell wall anchor domain-containing protein [Staphylococcus aureus] | 104 | 104 | 7% | 8.00E-24 | 49.06% | 66.00% | 4.00% | HDM8613333.1 |
| LP125_RS11130 | TPA: WxL domain-containing protein [Staphylococcus aureus] | 45.1 | 45.1 | 97% | 0.001 | 22.54% | 40.00% | 19.00% | HDL8983360.1 |
| LP125_RS11335 | mucus-binding protein [Staphylococcus aureus] | 184 | 457 | 33% | 1.00E-44 | 35.71% | 47.00% | 11.00% | MDU3924263.1 |
| LP125_RS11345 | No similarity |  |  |  |  |  |  |  |  |
| LP125_RS11385 | hypothetical protein V070_01369 [Staphylococcus aureus C0673] | 172 | 306 | 23% | 5.00E-44 | 38.75% | 57.00% | 2.00% | EZX22480.1 |
| LP125_RS11935 | No similarity |  |  |  |  |  |  |  |  |
| LP125_RS12380 | No similarity |  |  |  |  |  |  |  |  |
| LP125_RS01150 | hypothetical protein V070_01369 [Staphylococcus aureus C0673] | 58.9 | 58.9 | 46% | 1.00E-07 | 32.87% | 49.00% | 8.00% | EZX22480.1 |
| LP125_RS01490 | SdrD B-like domain-containing protein [Staphylococcus aureus] | 75.1 | 75.1 | 54% | 1.00E-11 | 26.74% | 57.00% | 12.00% | WP_210599654.1 |
| LP125_RS02875 | BspA family leucine-rich repeat surface protein [Staphylococcus aureus] | 142 | 142 | 60% | 6.00E-33 | 27.67% | 44.00% | 19.00% | MDF4035866.1 |
| LP125_RS13800 | No similarity |  |  |  |  |  |  |  |  |
| LP125_RS02315 | No similarity |  |  |  |  |  |  |  |  |
| LP125_RS02875 | BspA family leucine-rich repeat surface protein [Staphylococcus aureus] | 83.6 | 146 | 7% | 5.00E-14 | 38.33% | 56.00% | 8.00% | MDF4035866.1 |
| LP125_RS04395 | No similarity |  |  |  |  |  |  |  |  |
| LP125_RS06780 | mucus-binding protein [Staphylococcus aureus] | 212 | 956 | 51% | 2.00E-53 | 35.62% | 50.00% | 10.00% | MDU3924263.1 |
| LP125_RS07055 | surface exclusion protein SEA1/PrgA [Staphylococcus aureus] | 90.9 | 90.9 | 99% | 2.00E-16 | 25.23% | 40.00% | 20.00% | WP_176244423.1 |
| LP125_RS07170 | hypothetical protein V070_01369 [Staphylococcus aureus C0673] | 176 | 593 | 44% | 3.00E-45 | 40.43% | 57.00% | 3.00% | EZX22480.1 |
| LP125_RS08445 | No similarity |  |  |  |  |  |  |  |  |
| LP125_RS09470 | No similarity |  |  |  |  |  |  |  |  |
| LP125_RS09530 | No similarity |  |  |  |  |  |  |  |  |
| LP125_RS13040 | TPA: C40 family peptidase [Staphylococcus aureus] | 87.4 | 87.4 | 42% | 2.00E-17 | 41.67% | 57.00% | 6.00% | HDF0331430.1 |
| LP125_RS01700 | amidase domain-containing protein [Staphylococcus aureus] | 88.2 | 88.2 | 40% | 7.00E-16 | 28.13% | 58.00% | 1.00% | WP_072468802.1 |
| LP125_RS14880 | No similarity |  |  |  |  |  |  |  |  |
| LP125_RS05575 | N-acetylmuramoyl-L-alanine amidase [Staphylococcus aureus] | 289 | 289 | 89% | 2.00E-96 | 55.12% | 71.00% | 0.00% | WP_150530772.1 |
| LP125_RS08480 | No similarity |  |  |  |  |  |  |  |  |
| LP125_RS09840 | TPA: peptidase M23 [Staphylococcus aureus] | 114 | 114 | 19% | 9.00E-29 | 73.53% | 76.00% | 0.00% | HCV8089509.1 |
