# Supplementary material for: Lactobacilli-host interactions inhibit Staphylococcus aureus and Escherichia coli-induced cell death and invasion in a cellular model of infection
Source: Front Microbiol. 2024 Dec 18;15:1501119. doi: 10.3389/fmicb.2024.1501119 (PMC11688250; doi:10.3389/fmicb.2024.1501119)
Supplement: Supplementary file 3 [file Table_3.DOCX]

**Supplementary Table 3.** Sequence identity of cell-surface proteins encoded by L125 to proteins encoded by *E. coli.*

| ***Lp. plantarum L125* locus tag** | **Description** | **Max score** | **Total score** | **Query cover** | **E-value** | **Identity (%)** | **Positive substitutions (%)** | **Gaps (%)** | **Accesion** |
| --- | --- | --- | --- | --- | --- | --- | --- | --- | --- |
| LP125_RS10075 | TPA: fibrinogen-binding MSCRAMM adhesin Fss1 [Escherichia coli] | 81.3 | 221 | 30% | 1.00E-12 | 27.62% | 46.00% | 12.00% | HBD5769363.1 |
| LP125_RS10525 | peptide pheromone-binding protein TraC [Escherichia coli] | 350 | 350 | 93% | 1.00E-111 | 37.38% | 56.00% | 3.00% | MDU6989840.1 |
| LP125_RS11095 | No similarity |  |  |  |  |  |  |  |  |
| LP125_RS11130 | WxL domain-containing protein [Escherichia coli] | 63.2 | 63.2 | 82% | 1.00E-09 | 32.21% | 45.00% | 13.00% | MBE0792207.1 |
| LP125_RS11335 | BspA family leucine-rich repeat surface protein [Escherichia coli] | 184 | 515 | 18% | 2.00E-45 | 38.00% | 52.00% | 4.00% | MBL1007827.1 |
| LP125_RS11350 | BspA family leucine-rich repeat surface protein [Escherichia coli] | 219 | 831 | 60% | 2.00E-58 | 36.51% | 58.00% | 4.00% | MBL0960876.1 |
| LP125_RS11385 | MucBP domain-containing protein [Escherichia coli] | 146 | 677 | 35% | 8.00E-37 | 45.02% | 53.00% | 3.00% | MBC8929979.1 |
| LP125_RS11935 | No similarity |  |  |  |  |  |  |  |  |
| LP125_RS12380 | WxL domain-containing protein [Escherichia coli] | 53.9 | 53.9 | 17% | 4.00E-05 | 26.07% | 41.00% | 21.00% | MBE0792207.1 |
| LP125_RS01150 | MucBP domain-containing protein [Escherichia coli] | 52.8 | 52.8 | 39% | 9.00E-06 | 38.33% | 47.00% | 5.00% | MBC8921713.1 |
| LP125_RS01490 | collagen-binding protein [Escherichia coli] | 150 | 1928 | 42% | 1.00E-34 | 34.60% | 53.00% | 8.00% | PPI97406.1 |
| LP125_RS02875 | bacterial Ig-like domain-containing protein [Escherichia coli] | 93.6 | 162 | 19% | 7.00E-19 | 32.58% | 53.00% | 10.00% | WP_142440837.1 |
| LP125_RS13800 | autotransporter domain-containing protein [Escherichia coli] | 59.3 | 59.3 | 13% | 6.00E-06 | 36.77% | 47.00% | 3.00% | WP_201795598.1 |
| LP125_RS02315 | tail needle knob protein [Escherichia coli] | 48.5 | 48.5 | 14% | 0.004 | 33.59% | 54.00% | 7.00% | WP_181205478.1 |
| LP125_RS02875 | No similarity |  |  |  |  |  |  |  |  |
| LP125_RS04395 | No similarity |  |  |  |  |  |  |  |  |
| LP125_RS06780 | hypothetical protein [Escherichia coli] | 112 | 620 | 45% | 1.00E-24 | 35.98% | 55.00% | 3.00% | WP_270571553.1 |
| LP125_RS07055 | No similarity |  |  |  |  |  |  |  |  |
| LP125_RS07170 | MucBP domain-containing protein [Escherichia coli] | 153 | 679 | 49% | 3.00E-39 | 44.29% | 59.00% | 5.00% | MBC8929979.1 |
| LP125_RS08445 | No similarity |  |  |  |  |  |  |  |  |
| LP125_RS09470 | No similarity |  |  |  |  |  |  |  |  |
| LP125_RS09530 | TPA: collagen-binding MSCRAMM adhesin Scm [Escherichia coli] | 207 | 207 | 38% | 6.00E-57 | 39.23% | 59.00% | 6.00% | HCC5232179.1 |
| LP125_RS13040 | C40 family peptidase [Escherichia coli] | 119 | 119 | 46% | 2.00E-29 | 48.80% | 67.00% | 3.00% | MBE0791718.1 |
| LP125_RS01700 | TPA: glycoside hydrolase family 73 protein [Escherichia coli] | 94.7 | 94.7 | 18% | 8.00E-18 | 49.33% | 62.00% | 4.00% | HCC5232506.1 |
| LP125_RS14880 | C40 family peptidase [Escherichia coli] | 115 | 115 | 90% | 7.00E-27 | 32.84% | 56.00% | 8.00% | MBO9107039.1 |
| LP125_RS05575 | N-acetylmuramoyl-L-alanine amidase [Escherichia coli] | 95.1 | 95.1 | 61% | 3.00E-22 | 34.83% | 55.00% | 3.00% | WP_167399300.1 |
| LP125_RS08480 | TPA: C40 family peptidase [Escherichia coli] | 115 | 115 | 22% | 7.00E-25 | 48.31% | 61.00% | 5.00% | HCC5232428.1 |
| LP125_RS09840 | LysM peptidoglycan-binding domain-containing protein [Escherichia coli] | 110 | 162 | 54% | 1.00E-26 | 46.56% | 60.00% | 3.00% | MSH49934.1 |
