# Supplementary material for: Lactobacilli-host interactions inhibit Staphylococcus aureus and Escherichia coli-induced cell death and invasion in a cellular model of infection
Source: Front Microbiol. 2024 Dec 18;15:1501119. doi: 10.3389/fmicb.2024.1501119 (PMC11688250; doi:10.3389/fmicb.2024.1501119)
Supplement: Supplementary file 4 [file Table_4.DOCX]

**Supplementary Table 4.** Gene set enrichment analysis against the KEGG database for differentially enriched pathways in HT-29 cells treated with L125.

| **ID** | **GeneSet** | **NumGenes** | **p.adj** | **avg.logfc.dir** |
| --- | --- | --- | --- | --- |
| hsa04520 | Adherens junction | 72/72 | 2.28E-09 | -3.178815031 |
| hsa03040 | Spliceosome | 134/134 | 7.94E-05 | -3.291839128 |
| hsa05220 | Chronic myeloid leukemia | 71/71 | 6.26E-07 | -2.739479273 |
| hsa04919 | Thyroid hormone signaling pathway | 116/116 | 1.68E-05 | -3.107836928 |
| hsa04330 | Notch signaling pathway | 48/48 | 0.000683 | -3.298336924 |
| hsa05016 | Huntington's disease | 193/193 | 0.000713 | -2.980986218 |
| hsa00190 | Oxidative phosphorylation | 133/133 | 0.018484 | -2.619762343 |
| hsa00310 | Lysine degradation | 59/59 | 1.45E-05 | -3.595246805 |
| hsa05100 | Bacterial invasion of epithelial cells | 76/76 | 2.81E-05 | -2.835521119 |
| hsa05213 | Endometrial cancer | 50/50 | 6.49E-05 | -2.694403991 |
| hsa03015 | mRNA surveillance pathway | 91/91 | 9.06E-05 | -2.741768001 |
| hsa05212 | Pancreatic cancer | 64/64 | 9.10E-05 | -2.594226238 |
| hsa04152 | AMPK signaling pathway | 120/121 | 1.68E-05 | -3.00464182 |
| hsa05010 | Alzheimer's disease | 171/171 | 0.03318 | -2.861961965 |
| hsa04140 | Regulation of autophagy | 127/127 | 1.60E-05 | -2.080487232 |
| hsa04144 | Endocytosis | 260/260 | 0.000244 | -2.652371297 |
| hsa05205 | Proteoglycans in cancer | 203/203 | 0.00011 | -3.121229341 |
| hsa04810 | Regulation of actin cytoskeleton | 211/212 | 3.94E-05 | -3.408242148 |
| hsa05221 | Acute myeloid leukemia | 55/55 | 0.000289 | -2.985349993 |
| hsa04120 | Ubiquitin mediated proteolysis | 137/137 | 0.000224 | -2.921788873 |
