# Supplementary figures and images for: Lactobacilli-host interactions inhibit Staphylococcus aureus and Escherichia coli-induced cell death and invasion in a cellular model of infection
Source: Front Microbiol. 2024 Dec 18;15:1501119. doi: 10.3389/fmicb.2024.1501119 (PMC11688250; doi:10.3389/fmicb.2024.1501119)

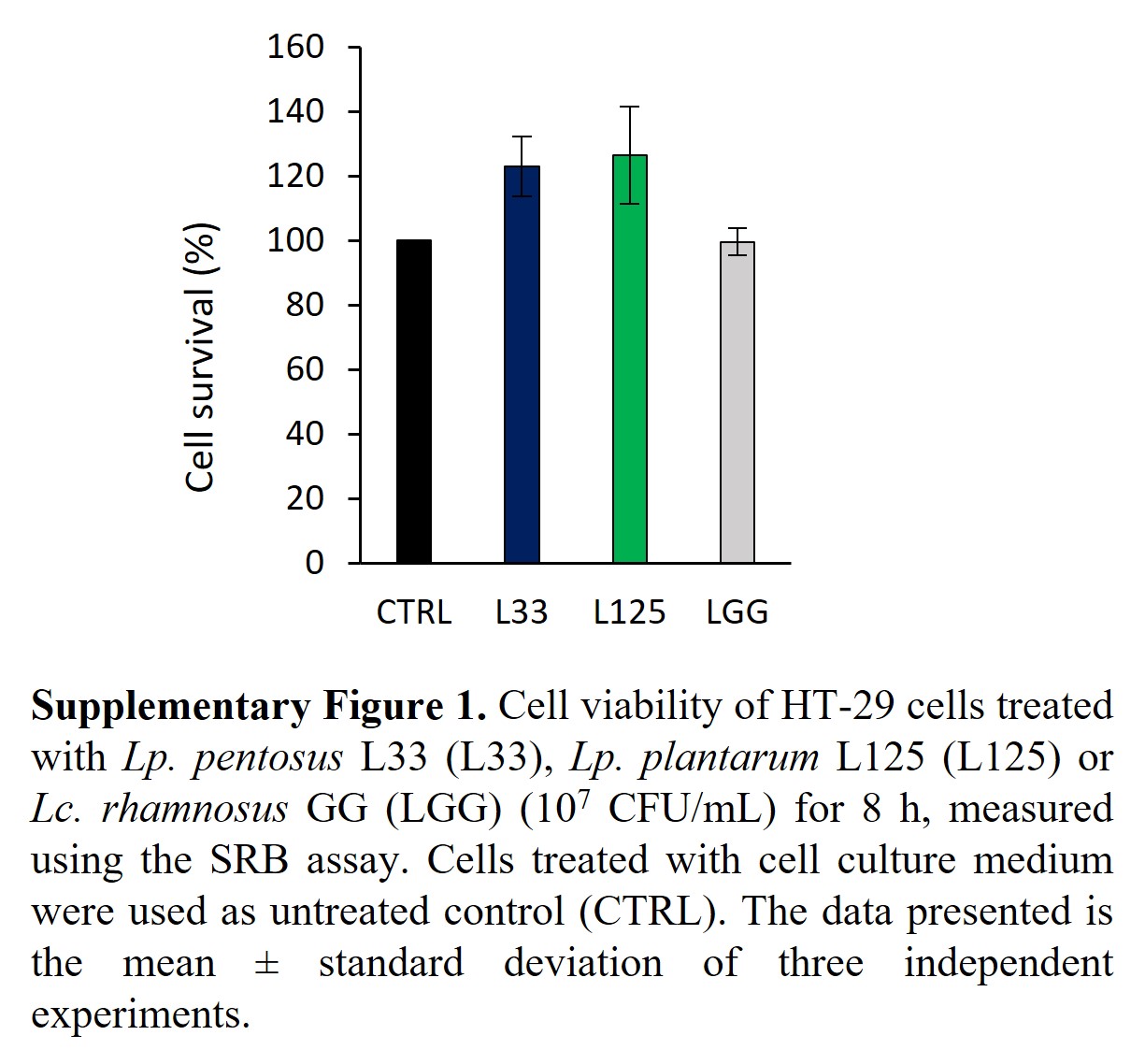

Supplement: Supplementary file 5 [file Image_1.JPEG]

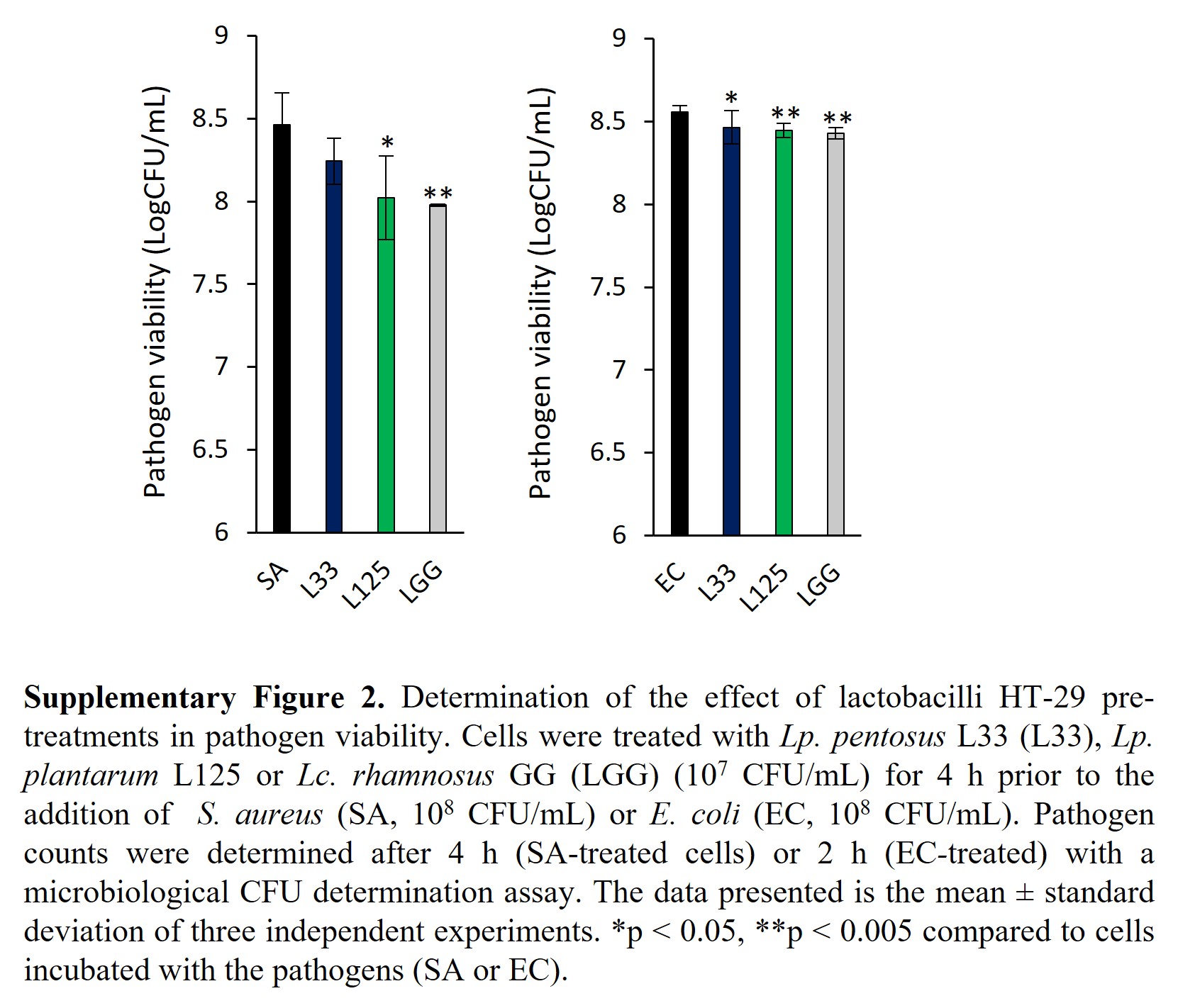

Supplement: Supplementary file 6 [file Image_2.JPEG]

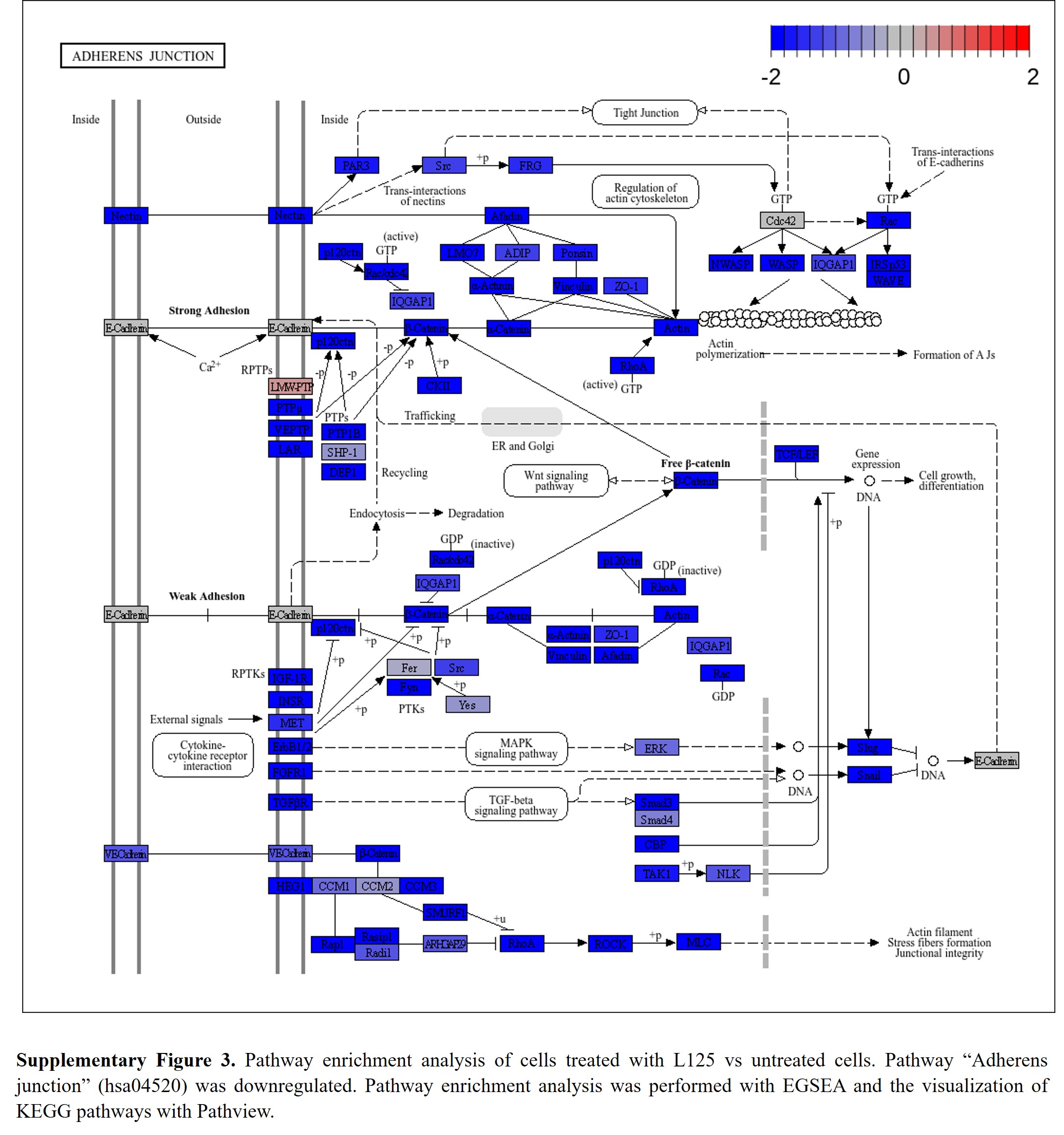

Supplement: Supplementary file 7 [file Image_3.JPEG]

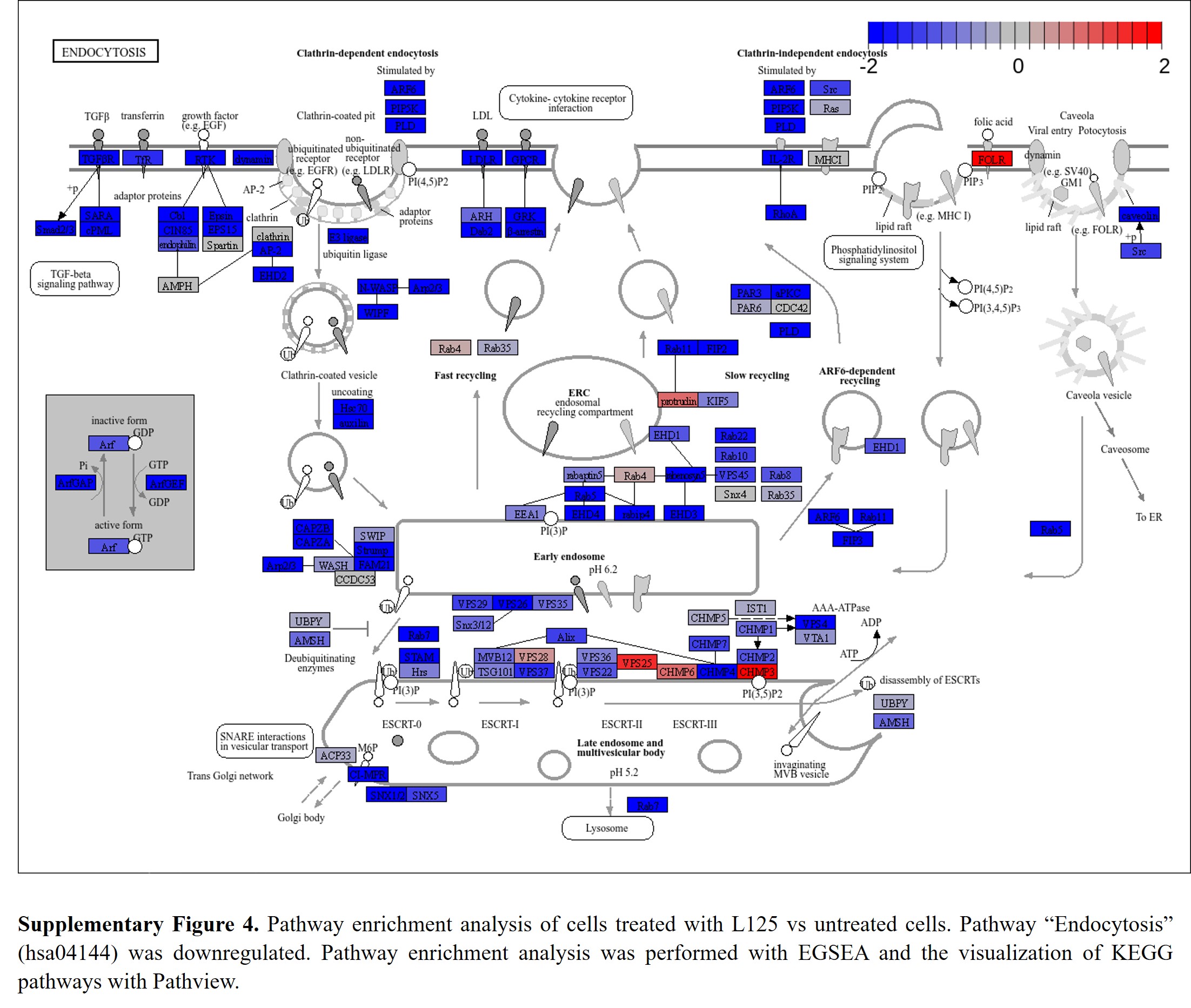

Supplement: Supplementary file 8 [file Image_4.JPEG]
